# Interaction between parental environment and genotype affects plant and seed performance in *Arabidopsis*

**DOI:** 10.1093/jxb/eru378

**Published:** 2014-09-18

**Authors:** Hanzi He, Deborah de Souza Vidigal, L. Basten Snoek, Sabine Schnabel, Harm Nijveen, Henk Hilhorst, Leónie Bentsink

**Affiliations:** ^1^Wageningen Seed Lab, Laboratory of Plant Physiology, Wageningen University, Droevendaalsesteeg 1, NL-6708 PB Wageningen,The Netherlands; ^2^Laboratory of Nematology, Wageningen University, Droevendaalsesteeg 1, NL-6708 PB Wageningen, The Netherlands; ^3^Biometris-Applied Statistics, Wageningen University and Research Centre, Droevendaalsesteeg 1, NL-6708 PB Wageningen, The Netherlands; ^4^Centre for BioSystems Genomics, Droevendaalsesteeg 1, NL-6708 PB Wageningen, The Netherlands; ^5^Laboratory of Bioinformatics, Wageningen University, Droevendaalsesteeg 1, NL-6708 PB Wageningen, The Netherlands; ^6^Department of Molecular Plant Physiology, Utrecht University, NL-3584 CH Utrecht, The Netherlands

**Keywords:** Genotypes, light, nitrate, parental environment, phosphate, plant performance, seed performance, temperature.

## Abstract

Seed performance after dispersal is highly dependent on parental environmental cues, especially during seed formation and maturation. Here we examine which environmental factors are the most dominant in this respect and whether their effects are dependent on the genotypes under investigation. We studied the influence of light intensity, photoperiod, temperature, nitrate, and phosphate during seed development on five plant attributes and thirteen seed attributes, using 12 *Arabidopsis* genotypes that have been reported to be affected in seed traits. As expected, the various environments during seed development resulted in changed plant and/or seed performances. Comparative analysis clearly indicated that, overall, temperature plays the most dominant role in both plant and seed performance, whereas light has a prominent impact on plant traits. In comparison to temperature and light, nitrate mildly affected some of the plant and seed traits while phosphate had even less influence on those traits. Moreover, clear genotype-by-environment interactions were identified. This was shown by the fact that individual genotypes responded differentially to the environmental conditions. Low temperature significantly increased seed dormancy and decreased seed longevity of NIL*DOG1* and *cyp707a1-1*, whereas low light intensity increased seed dormancy and decreased seed longevity of NIL*DOG3* and NIL*DOG6*. This also indicates that different genetic and molecular pathways are involved in the plant and seed responses. By identifying environmental conditions that affect the dormancy vs longevity correlation in the same way as previously identified naturally occurring loci, we have identified selective forces that probably shaped evolution for these important seed traits.

## Highlight text

The genotype-by-environment interactions of five parental environments with seed and plant performance are mediated by distinct genetic and molecular pathways, and the selective pressures that have shaped their natural variation.

## Introduction

Seed performance refers to the capacity of seeds to germinate under various environmental conditions and represents a critical component of the plant life cycle that is of eminent ecological and agronomic importance. It has been observed that a change of temperature, photoperiod, or nutrient or drought stress, during seed development, maturation, and after dispersal, may strongly affect seed performance (reviewed by Donohue, 2009). The principles of plasticity or adaptation of species with respect to seed performance in response to environmental changes is still largely unclear (Donohue *et al.*, 2005a; Donohue, 2009; Walck *et al.*, 2011). However, it is imperative to increase our knowledge as climate changes are expected to play a powerful and diverse role in ecosystems all over the world.

One of the characteristics that determines seed performance is seed dormancy. In natural environments dormancy of dry seeds can be released by storage of the seeds for several months at mild temperatures (after-ripening) or by cold stratification, which is a low-temperature treatment of imbibed seeds (Bewley *et al.*, 2013). The roles of temperature, light quality, photoperiod, water, and nutrients in determining the degree of seed dormancy have been investigated in a wide range of species (Fenner, 1991; Hilhorst, 1995; Holdsworth *et al.*, 2008; Bewley *et al.*, 2013). Seeds that develop at warmer temperatures are generally less dormant at maturity than those that develop at cooler temperatures, as described for *Beta vulgaris*, *Lactuca sativa*, *Amaranthus retroflexus*, wild oat (*Avena fatua*) (Fenner, 1991), wheat (Biddulph *et al.*, 2007), lettuce (Contreras *et al.*, 2009), weedy rice (Gu *et al.*, 2006), and *Arabidopsis* (Donohue *et al.*, 2008; Kendall *et al.*, 2011; Kendall and Penfield, 2012; Huang *et al.*, 2014). Low temperature increases abscisic acid (ABA) content during seed development in *Arabidopsis*; plants grown at 15°C had 2-fold higher ABA content as compared to those grown at 22°C, whereas gibberellic acid (GA) levels were reduced around 3-fold (Kendall *et al.*, 2011). Nutrition can also affect seed dormancy; Alboresi *et al.* (2005) showed that the depth of seed dormancy of *Arabidopsis* is inversely correlated with seed nitrate content. Higher nitrate concentrations (50mM) administered to the mother plant led to less dormant seeds than seeds produced under standard nitrate conditions (10mM). Nitrate probably affects seed dormancy by its effect on ABA synthesis and degradation, since Matakiadis *et al.* (2009) showed that increased endogenous nitrate led to lower ABA levels in *Arabidopsis* seeds.

In addition to an effect on seed dormancy, environmental cues during seed development can also affect other traits that contribute to seed performance, such as seed weight, seed yield, ability to germinate, and longevity (or ‘storability’). A recent study has shown that some of these traits are directly linked. Prevailing stress conditions, such as high salt, osmotic stress, high and low temperature, ABA treatment, and artificial ageing have a negative effect on germination, whereas seed size has a negative correlation with germination in the presence of ABA but a positive correlation with the rate of germination (Joosen *et al.*, 2012). Nguyen *et al.* (2012) demonstrated a negative correlation between seed dormancy and seed longevity (deeper seed dormancy correlated with shorter longevity and better longevity correlated with lower seed dormancy) for natural alleles of several *DELAY OF GERMINATION* (*DOG*) loci.

ABA is a major player in plant responses to various environmental stresses and this plant hormone is also thought to play a role in seed performance after environmental stress. ABA levels increase during seed maturation and in response to different abiotic stresses (Xiong and Zhu, 2003), including drought, high salinity, or low temperature (Iuchi *et al.*, 2001; Kendall *et al.*, 2011). The family of the 9-*cis*-epoxycarotenoid dioxygenases (*NCED*s) catalyse the first committed step in ABA biosynthesis and *NCED* genes are key elements in the control of ABA levels in seeds (Tan *et al.*, 2003; Lefebvre *et al.*, 2006). The *NCED* genes are involved in regulating key physiological processes in seeds, such as development, maturation, desiccation, and germination, by affecting the ABA concentration (Iuchi *et al.*, 2001; Tan *et al.*, 2003; Lefebvre *et al.*, 2006). In *Arabidopsis*, *NCED3* expression is induced by drought stress and the endogenous ABA content under drought stress is increased, thereby increasing seed dormancy (Iuchi *et al.*, 2001; Frey *et al.*, 2012). *NCED6* and *NCED9* have been shown to be essential for ABA production in the embryo and endosperm that imposes dormancy, whereas *NCED5* fine-tunes ABA accumulation and affects seed dormancy and drought tolerance together with other *NCED* family members (Frey *et al.*, 2012). Members of the *CYP707A* (Cytochrome P450, Family 707, Subfamily A) gene family, which catalyse steps of the ABA catabolic pathway, also play a prominent role in regulating endogenous ABA levels during seed development and germination (Okamoto *et al.*, 2006). *CYP707A* transcript levels increased in response to abiotic stress, dehydration, and exogenous ABA treatment (Saito *et al.*, 2004). *CYP707A1* is expressed predominantly during mid-maturation and is downregulated during late maturation, whereas *CYP707A2* transcript levels increase from late maturation to the mature dry seed stage, indicating that *CYP707A2* plays a major role in reducing the ABA content in after-ripening *Arabidopsis* seeds or during early seed imbibition (Kushiro *et al.*, 2004; Okamoto *et al.*, 2006; Matakiadis *et al.*, 2009). Seeds of T-DNA insertion mutants of *CYP707A2* have a higher ABA content and exhibit increased dormancy, as compared to wild-type plants (Kushiro *et al.*, 2004).

Here, we investigated which parental environment most strongly affected seed and plant performance and whether there are genotype-by-environment interactions. We used different genotypes: a set of *DOG* near-isogenic lines (*DOG*-NILs; Bentsink *et al.*, 2010) that are known to be affected in both dormancy and seed longevity (Nguyen *et al.*, 2012) levels by different genetic and molecular pathways, and several mutants that are defective in *DOG1* gene expression (Bentsink *et al.*, 2006) or ABA biosynthesis (*NCED6* and *NCED9*; Lefebvre *et al.*, 2006) and catabolism genes (*CYP707A1* and *CYP707A2*; Kushiro *et al.*, 2004; Saito *et al.*, 2004). These different genotypes might give a first indication of the genetic and molecular pathways that are involved in the response to the parental environment. A noticeable difference between mutants and NILs is that the genetic variation present in the NILs is the result of adaptations to local environmental variables. Seeds of all genotypes were harvested from plants grown under various light intensities, photoperiod, temperatures, and nitrate and phosphate concentrations, and seed performance was analysed by the after-ripening requirement to release seed dormancy, seed longevity, and germination under several stress conditions. In this paper we show that interactions between parental environment and genotype clearly affect plant and seed performance in *Arabidopsis*.

## Materials and methods

### Plant materials

The *Arabidopsis thaliana* accessions Landsberg *erecta* (L*er*-0, L*er*), Columbia (Col-0, Col) and other genotypes with the L*er* and Col genetic backgrounds were used in this study. NIL*DOG1*-Cvi (Cape Verde Islands), NIL*DOG2-*Cvi, NIL*DOG3*-Cvi, NIL*DOG6*-Kas-2 (Kashmir), NIL*DOG22*-An-1 (Antwerpen) (Alonso-Blanco *et al.*, 2003; Bentsink *et al.*, 2010), and the *dog1-1* mutant (Bentsink *et al.*, 2006) are lines with a L*er* genetic background, whereas *dog1-3* (SALK 000867, T-DNA insertion in the promoter region of *DOG1*) (Bentsink *et al.*, 2006), *cyp707a1-1*, *cyp07a2-1* (Kushiro *et al.*, 2004), and the *Atnced6-Atnced9* double mutant (Lefebvre *et al.*, 2006) are lines with a Col genetic background. Dormancy and longevity phenotypes have been described in the references above.

### Growth conditions

Seeds were sown in petri dishes on water-soaked filter paper followed by a 4-d cold treatment at 4°C, and transferred to a climate room at 22°C with continuous light for 3 d before planting. Germinated seedlings were grown on 4×4cm Rockwool blocks in a growth chamber at 20°C/18°C (day/night) under a 16-h photoperiod of artificial light (150 μmol m^–2^ s^–1^) and 70% relative humidity. Plants were grown in a standard nutrient solution (Supplementary Table S1) and watered three times per week. Upon the start of flowering, plants were transferred to the various environmental conditions (Table 1); for each condition there were three biological replicates containing five plants per replicate.

**Table 1.** Environmental conditions before and after the start of flowering^a^

Environmental factor	Before flowering	After flowering
Light Intensity	Standard (SL)	Low (75 μmol m^–2^ s^–1^) (LL)
		Standard (150 μmol m^–2^ s^–1^) (SL)
		High (300 μmol m^–2^ s^–1^) (HL)
Photoperiod	Long day (LD)	Short Day (8h daylength) (SD)
		Long Day (16h daylength) (LD)
		Continuous light (CL)
Temperature	20°C	15°C
		20°C
		25°C
Nitrate	5mM (N5)	0mM (N0)
		5mM (N5)
		20mM (N20)
Phosphate	0.5mM, 20°C (P0.5_20°C)	0.01mM, 20°C (P0.01_20°C)
		0.5mM, 20°C (P0.5_20°C)
		3mM, 20°C (P3_20°C)
	0.5mM, 25°C (P0.5_25°C)	0.01mM, 25°C (P0.01_25°C)
		0.5mM, 25°C (P0.5_25°C)
		3mM, 25°C (P3 _25°C)

^a^ After the start of flowering the plants were transferred to different parental conditions. For each condition the abbreviation that is used in Figs 1–5 has been indicated within brackets. Standard light intensity (SL), long days (LD), 20°C, 5mM nitrate (N5), and 0.5mM phosphate (P0.5) were regarded as control conditions.

Plants that were known to flower earlier (NIL*DOG2* and NIL*DOG22*) were planted 5 d later in order to synchronize the flowering. In case individual plants had already started flowering, those flowers and siliques were removed to make sure all the seeds developed under the specific environmental conditions. Due to space limitation in the growth compartments each environment was performed as an independent experiment containing the control condition, except for the second nitrate and temperature experiment as these were performed at the same time and therefore share the control. All experiments with each growth condition were executed twice (first and second growth) for a robust confirmation of the phenotypes.

### Plant phenotyping

Plant height, number of siliques per plant, and number of seeds per silique were scored for all three replicates. To investigate the number of seeds per silique and seed size, flowers that had opened at day 10 after the start of flowering were tagged and corresponding seeds were harvested. When the majority of siliques had turned yellow, watering of the plants was stopped and 7 d later dry mature seeds were harvested (Huang *et al.*, 2014). These criteria were used for harvesting seeds in all the environments. The number of seeds was determined by taking photographs of the seeds on white filter paper (20.2×14.3cm white filter paper, Allpaper BV, Zevenaar, The Netherlands; http://www.allpaper.nl) using a Nikon D80 camera fixed to a repro stand with a 60mm macro objective. The camera was connected to a computer with Nikon Camera Control Pro software version 2.0. Clustering of seeds was prevented as much as possible. The photographs were analysed using ImageJ (http://rsbweb.nih.gov/ij/) by combining colour thresholds (Y_100–255_U_0–85_V_0–255_) with particle analysis.

### Seed phenotyping

Seeds were harvested in bulk from five plants. Seeds were weighed with an AD-4 autobalance (PerkinElmer, Inc.). Single seed weight was determined by weighing around 5mg of seeds, divided by the number of the weighed seeds, and converted to a 1000-seed weight by multiplying by 1000.

Germination experiments were performed as described previously (Joosen *et al.*, 2010). In brief, two layers of blue germination paper were equilibrated with 48ml demineralized water in plastic trays (15×21cm). Six samples of approximately 50 to 150 seeds were spread on wetted papers using a mask to ensure accurate spacing. Piled up trays were wrapped in a closed transparent plastic bag. The experiment was carried out in a 22°C incubator under continuous light (143 μmol m^–2^ s^–1^). Pictures were taken twice a day for a period of 6 d using the same camera and software as described for number of seeds.

Germination was scored using the Germinator package (Joosen *et al.*, 2010). To quantify seed dormancy (DSDS50: days of seed dry storage required to reach 50% germination), germination tests were performed weekly until all seed batches had germinated by >90%. A generalized linear model with a logit link as described by Hurtado *et al.* (2012) was adapted to calculate DSDS50. Germination data were adjusted by choosing *n* = 100 and fitted as one smooth curve per line. The observed germination proportion was re-interpreted as having observed y ‘successes’ in *n* binomial trials (e.g. 75% germinated means y = 75 out of 100 possible ‘trials’). DSDS50 is the closest time point to where a horizontal line at y = 50 crosses the fitted curve.

Germination under stress conditions was performed on fully after-ripened seeds. Stress conditions were: temperature stress (10°C, 30°C); osmotic stress (–0.8MPa mannitol; Sigma-Aldrich); salt stress (125mM NaCl; Sigma-Aldrich); and ABA stress (0.2 µM ABA; Duchefa Biochemie). ABA was dissolved in 10mM MES buffer (Sigma-Aldrich) and the pH adjusted to 5.8. To measure seed longevity, an artificial ageing test was performed by incubating seeds above a saturated ZnSO_4_ solution (40°C, 85% relative humidity) in a closed tank with circulation for 5 d (ISTA, 2012). In the accelerated aging method (ISTA, 2012) and in our artificial ageing method the seeds are constantly incubated in the same relative humidity combined with a warm temperature. The accelerated ageing method of ISTA uses near-100% relative humidity, whereas we used 85% relative humidity. The seeds were then taken out and germinated on demineralized water as described previously.

### Germination parameters

Maximum germination (*G*_*max*_) values were extracted from the germination assay using the Germinator package (Joosen *et al.*, 2010). *G*_*max*_ is the final germination percentage at the end of the germination assay. For germination in demineralized water (control), and germination at 10°C the *G*_*max*_ of most genotypes reached 100%. Therefore, to better distinguish the small differences between genotypes, the rate of germination (*t*_*50*_: the time required to reach 50% germination of the total number of germinated seeds) was also used for data analysis.

### Nitrate, phosphate, and phytate determinations

To measure nitrate, phosphate, and phytate content, 5mg of seeds were boiled at 100°C for 15min in 0.5ml 0.5M HCl and 50mg l^–1^ *trans*-aconitate (internal standard). After centrifuging for 2min at 13 000rpm, 200 µl of the supernatant was transferred to an HPLC-vial.

HPLC-analysis was performed on a Dionex ICS2500 system with an AS11-HC column and an AG11-HC guard column and eluted with NaOH. The elution procedure was: 0–15min linear gradient of 25–100mM NaOH, then 15–20min 500mM NaOH followed by 20–35min 5mM NaOH. Flow rates were 1ml min^–1^ throughout the run. Contaminating anions in the eluents were removed using an ion trap column (ATC), installed between the pump and the sample injection valve. Anions were determined by conductivity detection. Background conductivity was decreased using an ASRS suppressor, with water as a counterflow. Peaks were identified and quantified using known external standards. External standards of nitrate, phosphate, and phytate were NaNO_3_ (Merck), Na_2_HPO_4_.2H_2_O (Merck), and Na(12)-IP6 IP6 (Sigma-Aldrich), respectively.

### Data analysis

All data (both first and second growth) analysis was done in the statistical programming environment R 3.0.0.

#### 

##### Integrated analysis of all factors contributing to plant and seed performance: 

All data were analysed together. For comparison between traits each trait data set was normalized to a scale of 0–100. Analysis of variance (ANOVA) using linear models was used to determine the significance of the different environmental variables one by one.

##### Integrated analysis of the effect of seed maturation environments on each plant and seed performance: 

The data set was split up by environmental factors as shown in Table 1. For data generated in each environment a linear model was fitted to determine the significance of the variable within the set. This was done over all genotypes. The significance threshold was adjusted for multiple testing by using significance 0.05 dividing the number of test (95) (*P* = 0.000526).

##### Trait-by-trait correlation/significance of plant and seed performance: 

All data were used for this investigation. Pearson correlation was calculated for all trait pairs and significance was determined by linear regression.

##### Genotype-by-environment interactions: 

Data were split by environmental factors as described in Table 1. Genotype-by-environment interaction was determined by ANOVA using a linear model (trait~environment*genotype). Boxplots were generated by the standard R boxplot function, using the same linear model and data as use in the ANOVA as input.

## Results

To identify the parental environment with the most dominant effect on seed performance, the different genotypes from flowering onwards were grown in the environments listed in Table 1. 12 genotypes were used, including two wild-type accessions, Landsberg *erecta* (L*er*) and Columbia (Col-0), five near-isogenic lines (NIL*DOG1*, NIL*DOG2*, NIL*DOG3*, NIL*DOG6*, and NIL*DOG22*) that are known to affect seed dormancy (Bentsink *et al.*, 2010), as well as two mutants that are affected in the *DOG1* gene (*dog1-1* and *dog1-3*) and three mutants of ABA biosynthesis and catabolism genes (*cyp707a1-1*, *cyp707a2-1* and *nced6 nced9*). Here, seed performance refers to the capacity of seeds to germinate under varying environmental conditions. Several seed germination traits were determined, including seed dormancy (DSDS50), seed longevity, and germination under stress conditions (i.e. high and low temperatures, osmotic stress, salt stress, and ABA stress conditions). The seed performance phenotypes observed in control conditions confirmed those that had been reported previously (Supplementary Figure S1) (Alonso-Blanco *et al.*, 2003; Kushiro *et al.*, 2004; Bentsink *et al.*, 2006; Lefebvre *et al.*, 2006; Bentsink *et al.*, 2010). Since the environment has both direct and indirect effects on seed performance we also monitored several plant phenotypes, such as the time that is required for seed maturation, plant height, number of siliques per plant, number of seeds per silique, seed size, and seed weight.

### The effect of the parental environment on the seed reproductive period

**Fig. 1.**
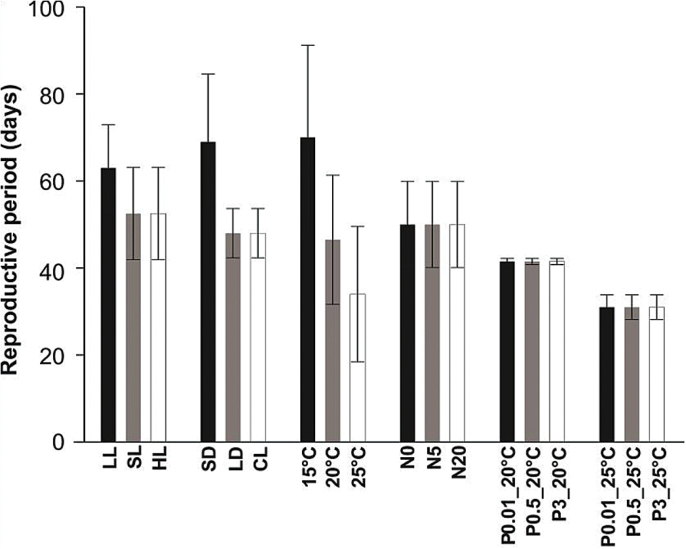
Plant reproductive periods in different environments as presented in Table 1. The average value of first and second growth was shown. Error bars show standard deviation. LL, low light; SL, standard light; HL, high light; SD, short days; LD, long days; CL, continuous light; N0, 0mM nitrate; N5, 5mM nitrate; N20, 20mM nitrate; P0.01_20°C, 0.01mM phosphate at 20°C; P0.5_20°C, 0.5mM phosphate at 20°C; P3_20°C, 3mM phosphate at 20°C; P0.01_25°C, 0.01mM phosphate at 25°C; P0.5_25°C, 0.5mM phosphate at 25°C; (P3_25°C, 3mM phosphate at 25°C. SL, LD, 20°C, N5, and P0.5 are control conditions.

Low light intensity and short days extended the seed reproductive period by ~10 d (Fig.1). In contrast, increased light intensity and extended photoperiod had no influence on the length of the seed reproductive period.

Low temperature (15°C) retards plant growth and, as a result, extends the reproductive period (Fig. 1). At 15°C, all genotypes required almost one month extra to complete their life cycle as compared to 20°C, whereas at higher temperature (25°C) the reproductive period was shortened by 12 d.

Different nitrate and phosphate concentrations had no effect on the reproductive period. In addition, the combination of phosphate and high temperature shortened the reproductive period as for high temperature alone. This confirmed that changes in phosphate level do not affect the length of the reproductive period.

### Generalized effects of the parental environments on plant and seed performance

Overall we have phenotyped 12 different genotypes (three biological replicates each) for 18 traits in 13 different environments and all these experiments have been performed twice. In order to assess and compare the importance of the different environmental factors on the phenotypes investigated, we performed statistical analysis on all data generated.

#### Relationships between traits

**Fig. 2.**
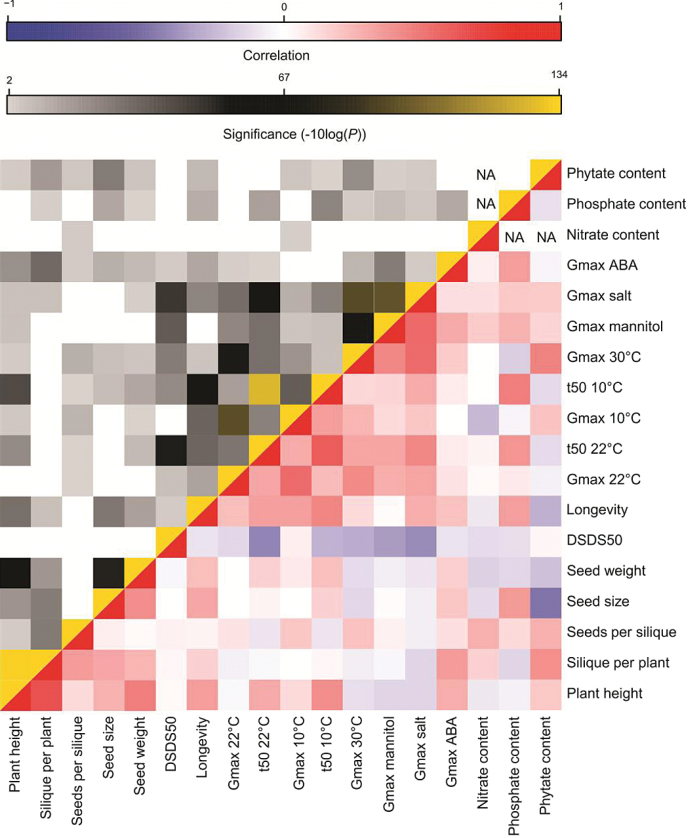
Trait by trait correlation/significance of plant and seed performance. The linear model was used to calculate all pairwise correlations between plant and seed performance traits. In the red/blue-coloured area, rectangles represent Pearson correlation coefficient r values (see correlation colour key). In the yellow/grey-coloured area, rectangles represent –log (*P*-values) of the Pearson correlation coefficients (see significance colour key) and empty rectangles represent not significant (*P*-values greater than 0.01). DSDS50 represents dormancy levels. Longevity is measured by artificial aging (40°C, 85% relative humidity). *G*_*max*_ is the final germination percentage at the end of the germination assay. *t*_*50*_ is the rate of germination. NA, not available.

A correlation matrix was generated for all pairs of measured traits to investigate associations between the characterized traits (Fig. 2 and Supplementary Table S2). The plant performance traits plant height and number of siliques per plant showed a strong and highly significant positive correlation. Plant height was also strongly correlated with seed weight, and, to a lesser extent, with seed size. Seed weight and seed size were strongly correlated. In general stress germination traits correlated with each other, especially germination in mannitol and salt, probably because both confer osmotic stress.

#### The effect of the parental environmental factors

**Fig. 3.**
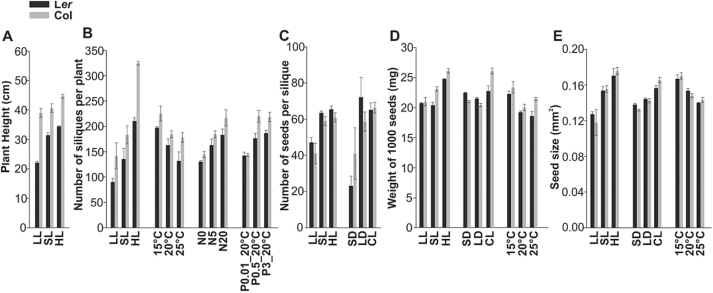
General effect of the seed maturation environment on plant performance. Plant performance of both Landsberg *erecta* (L*er*) and Columbia (Col) are presented: (A) plant height (cm), (B) number of siliques per plant, (C) number of seeds per silique (D) weight of 1000 seeds (mg), (E) seed size (mm^2^) for light intensity [low light (LL), standard light (SL), and high light (HL)], photoperiod [short day (SD), long day (LD), and continuous light (CL)], temperature (15, 20, and 25°C), nitrate concentrations (N0, N5, and N20) and phosphate concentrations (P0.01_20°C, P0.5_20°C, ad P3_20°C), respectively. Only the results that were significant (*P* < 0.000526; Table 3) and repeatable in both growths are presented here. Averages of three replicates are displayed. Error bars show standard errors.

**Fig. 4.**
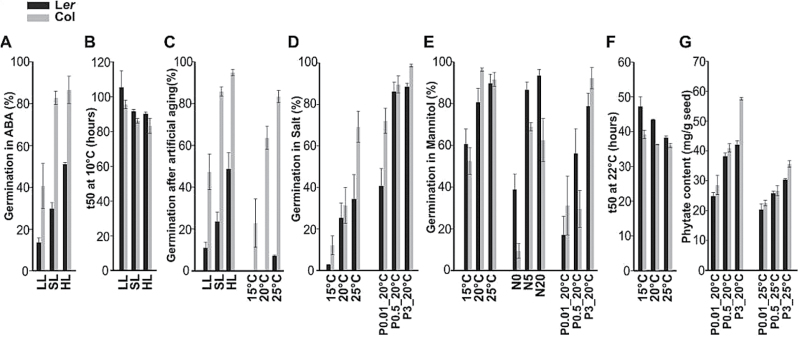
General effect of the seed maturation environment on seed performance. Seed performance of both Landsberg *erecta* (L*er*) and Columbia (Col) is presented: (A) germination in ABA (0.2 µM), (B) *t*_*50*_ for germination at low temperature (10°C), (C) germination after artificial ageing (40°C, 85% relative humidity), (D) germination in salt (125mM NaCl), (E) germination in mannitol (–0.8MPa), (F) *t*_*50*_ for germination at 22°C, (G) phytate content in seeds (mg g^–1^ seeds) for light intensity [low light (LL), standard light (SL), and high light (HL)], photoperiod [short days (SD), long days (LD), and continuous light (CL)], temperature (15, 20, and 25°C), nitrate concentrations (N0, N5, and N20) and phosphate concentrations × temperature (P0.01_20°C, P0.5_20°C, P3_20°C, P0.01_25°C, P0.5_25°C, and P3_25°C). Only the results that were significant (*P* < 0.000526; Table 3) and repeatable in both growths are presented here. Averages of three replicates are displayed. Error bars show standard errors.

We used linear models/ANOVA to investigate the overall variance caused by the different parental environments. This analysis revealed that in general the effect of the genotype was the most pronounced (*P <* 1×10^–200^) (Table 2), with a very prominent contribution of the genetic background (L*er* or Col) of the genotypes (*P <* 1×10^–113^). Also the effect of the parental environment was very significant (*P <* 1×10^–157^). Of the environmental factors, temperature had the most significant effect on the traits measured (*P <* 1×10^–60^; Table 2). A linear model was used to determine the significance of the different environments on each plant as well as on the seed traits (Table 3). Temperature played a dominant role in both plant and seed traits (as was also shown in Table 2), whereas light signals (light intensity and photoperiod) had more impact on plant traits. Nitrate mildly affected some of the plant and seed traits while phosphate had even less influence on those traits. To illustrate the direction of the effect of the parental environment, we have presented plant and seed performances in Fig. 3 and Fig. 4, respectively. Only the data for L*er* and Col are presented since most of the effects were similar in all genotypes and the largest differences were caused by the genetic background. Data for all the genotypes on two growths is available in Supplementary Figure S2. The significance of all the genotypes for both growths is shown in Table 3. The effects that are different from the control treatment in both independent growths (t-test, *P* < 0.05) are presented in Fig. 3 and Fig. 4.

**Table 2.** Integrated analysis of all factors contributing to plant and seed performance

Factor	*P*
Genotype	<1×10^–200^
Environmental summary^a^	<1×10^–157^
Background	<1×10^–113^
Temperature	<1×10^–60^
Light intensity	<1×10^–49^
Photoperiod	<1×10^–27^
Phosphate	<1×10^–25^
Nitrate	<1×10^–4^

^a^Environmental summary is the combination of the five environmental factors. For every factor the *P*-value is reported to indicate significance.

**Table 3.** Integrated analysis of the effect of seed maturation environments on plant and seed performance^a^

–10 log(*P*)	Light intensity	Photoperiod	Temperature	Nitrate	Phosphate	Phosphate × Temperature
Plant height	7.60	11.38	n.s.	n.s.	n.s.	n.s.
Silique per plant	24.63	24.79	10.00	4.88	6.35	n.s.
Seeds per silique	15.87	33.37	10.01	n.s.	n.s.	n.s.
Seed size	5.75	39.01	32.93	n.s.	4.08	n.s.
Seed weight	42.54	15.84	8.61	n.s.	n.s.	n.s.
DSDS50	n.s.	n.s.	7.39	3.65	n.s.	n.s.
Longevity	12.05	n.s.	16.78	n.s.	n.s.	n.s.
*G*_*max*_ 22°C	n.s.	n.s.	n.s.	n.s.	n.s.	n.s.
*t*_*50*_ 22°C	n.s.	n.s.	27.56	6.05	n.s.	n.s.
*G*_*max*_ 10°C	n.s.	n.s.	n.s.	n.s.	n.s.	n.s.
*t*_*50*_ 10°C	5.67	n.s.	8.02	5.28	n.s.	n.s.
*G*_*max*_ 30°C	n.s.	n.s.	13.77	n.s.	n.s.	n.s.
*G*_*max*_ mannitol	n.s.	n.s.	11.63	4.09	3.68	n.s.
*G*_*max*_ salt	n.s.	n.s.	11.88	n.s.	4.85	n.s.
*G*_*max*_ ABA	15.06	n.s.	6.64	n.s.	n.s.	n.s.
Nitrate content	n.a.	n.a.	n.a.	5.72	n.a.	n.a.
Phosphate content	n.a.	n.a.	n.a.	n.a.	n.s.	8.98
Phytate content	n.a.	n.a.	n.a.	n.a.	23.39	8.08

^a^ –10 log(*P*) values demonstrate significance levels. n.a., not available; n.s., not significant (*P* < 0.000526). DSDS50 represents dormancy levels. Longevity is measured by artificial aging (40°C, 85% relative humidity). *G*_*max*_ is the final germination percentage at the end of the germination assay. *t*_*50*_ is the rate of germination.

#### The effect of light intensity on plant and seed performance

Light intensity affected all the plant phenotypes significantly (Table 3). In high light intensity plants grew taller (Fig. 3A) and produced more siliques per plant (Fig. 3B). Low light intensity significantly decreased the number of seeds per silique (Fig. 3C). High light intensity resulted in heavier (Fig. 3D) and larger seeds (Fig. 3E). High light intensityalso had a positive effect on germination percentage in ABA (Fig. 4A), germination rate in 10°C (Fig. 4B), and seed longevity (Fig. 4C), as measured by artificial aging.

#### The effect of photoperiod on plant and seed performance

Both photoperiod and light intensity are important light signals for plant performance, but they play distinct roles. Short days decreased the number of seeds per silique (Fig. 3C) while continuous light resulted in heavier (Fig. 3D) and larger seeds (Fig. 3E). These results were in agreement with Contreras *et al.* (2008). Contrary to light intensity, photoperiod did not have any significant effect on seed performance (Table 3).

#### The effect of temperature on plant and seed performance

Low temperature (15°C) during seed maturation resulted in yield increases. Plants had more siliques (Fig. 3B) that contained heavier (Fig. 3D) and larger seeds (Fig. 3E). However, it is worth noting that the quality of these seeds was lower than that of the control, which is especially reflected in the decreased seed longevity (Fig. 4C), and decreased germination in salt (Fig. 4D) and in mannitol (Fig. 4E). The low seed maturation temperature also slowed the germination rate at 22°C (Fig. 4F).

#### The effect of nutrition on plant and seed performance

The effect of both nitrate and phosphate and a combination of phosphate regimes and high temperature on plant and seed performance was studied. Plants grown in the higher nitrate regime (20mM) produced more siliques (Fig. 3B). With respect to seed performance, low nitrate (0mM) decreased germination rate (Fig. 4B) and decreased germination in mannitol (Fig. 4E) but higher nitrate did not have a significant effect.

Phosphate levels correlated positively with the number of siliques per plant (Fig. 3B) (Zhao *et al.*, 2008; Dick *et al.*, 2011). Increasing phosphate content increased germination in stress conditions (Fig. 4D, E) at 20°C. Phytate is the main storage form of phosphate in dry seeds, and the level of phytate increased in the high phosphate maturation environment accordingly (Fig. 4G).

### Genotype-specific effects of the parental environment on seed performance

#### Genotype-by-environment interactions

**Fig. 5.**
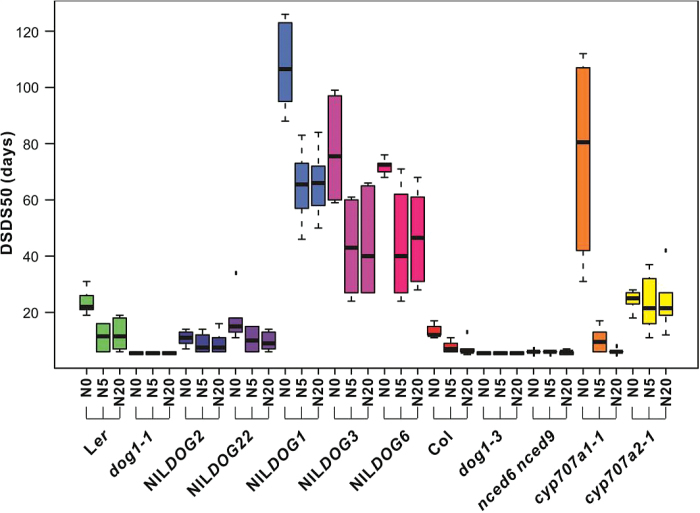
Genotype-by-environment interactions for seed dormancy behaviour after seed maturation in different nitrate regimes. The Boxplot presents dormancy levels (DSDS50) of 12 genotypes in three nitrate environmental conditions (N0, N5, and N20). The genotype-by-environment interaction is significant (*P* = 4.03^−18^).

Maturation environments have a noteworthy influence on seed dormancy levels, as well as on other plant and seed performance traits. However, several highly significant genotype-by-environment interactions suggest that the phenotypic plasticity varied among the 12 genotypes tested. All the significant (*P <* 0.001) genotype-by-environment interactions for the parental maturation environments are listed in Table 4. To visualize this genotype-by-environment effect we have shown the dormancy levels (DSDS50) for the nitrate environment (Fig. 5). Genotypes with higher primary dormancy levels display higher plasticity in the different nitrate environments. Thus, specific genetic regions (NILs) or genes (mutants) are the likely causal factors of higher plasticity. Furthermore, we see mainly an effect of reduced nitrate (0mM) and not of increased nitrate. Apparently, the nitrate response is saturated between N20 and N5. All of the significant genotype-by-environment interactions affecting plant and seed performances in the different environments are shown in Supplementary Figure S1. To explore this in more detail, we focus on genotype-specific effects in the following section.

**Table 4.** Significant genotype-by-environment interactions affecting plant and seed performance in all five environments^a^

Environment	Plant/Seed performance	G × E *P*-value
Light intensity	DSDS50	2.5^–10^
	*G*_*max*_ 10°C	3.3^–23^
	*G*_*max*_ 22°C	4.7^–15^
	*G*_*max*_ mannitol	2.2^–08^
	*G*_*max*_ salt	4.0^–09^
	Seed weight	3.8^–07^
Photoperiod	DSDS50	2.3^–09^
	Seed weight	2.2^–07^
Temperature	DSDS50	7.1^–09^
	*G*_*max*_ 22°C	3.1^–09^
	*G*_*max*_ 30°C	2.7^–04^
	*G*_*max*_ mannitol	7.6^–04^
	*G*_*max*_ salt	2.7^–05^
	Longevity	1.1^–13^
Nitrate	DSDS50	4.0^–18^
	*G*_*max*_ 10°C	8.7^–04^
	*G*_*max*_ 22°C	5.6^–13^
	*G*_*max*_ ABA	5.6^–04^
	*G*_*max*_ mannitol	2.2^–06^
Phosphate	*G*_*max*_ 30°C	1.4^–04^
	*G*_*max*_ salt	6.0^–04^

^a^ G × E, genotype-by-environment interaction. DSDS50 represents dormancy levels. Longevity is measured by artificial aging (40°C, 85% relative humidity). *G*_*max*_ is the final germination percentage at the end of the germination assay.

#### Effect of the parental environment on seed dormancy and longevity

**Fig. 6.**
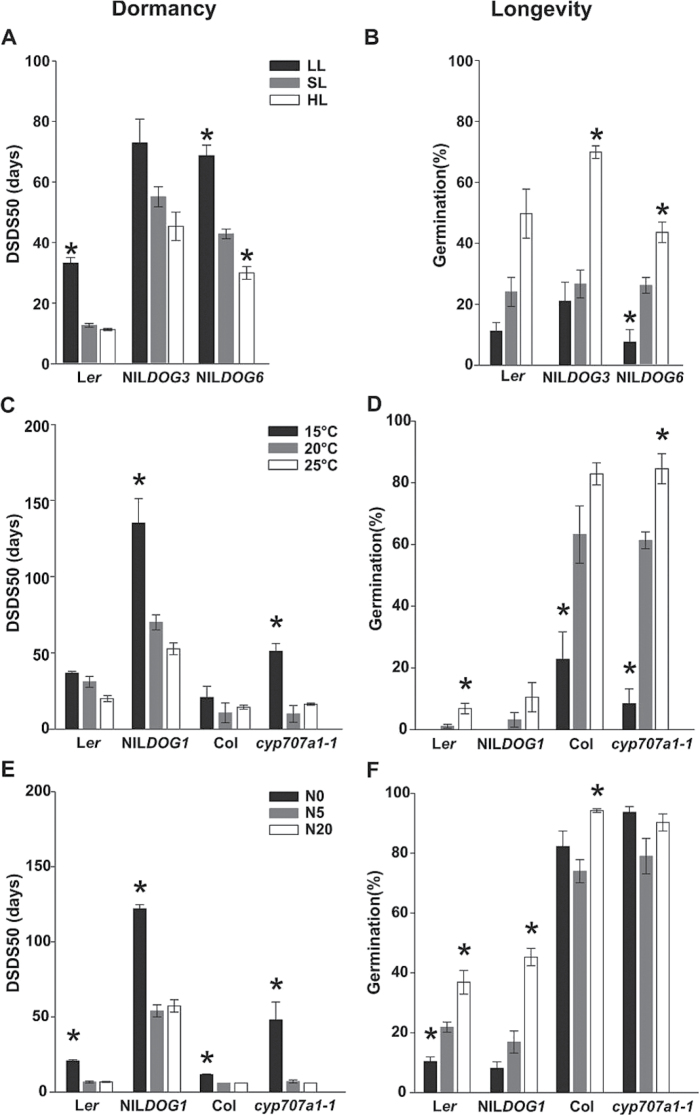
Dormancy (DSDS50) and longevity levels (germination after artificial aging) of seeds matured in different light intensity (A, B), temperature (C, D), and nitrate concentrations (E, F). Averages of three replicates are presented. Error bars show standard errors. Asterisks indicate significant differences between the treatment and control for each genotype (*P <* 0.05).

Low light intensity increased seed dormancy of NIL*DOG3* and NIL*DOG6* (Fig. 6A). Germination percentage after artificial ageing increased in low light intensity, indicating a negative correlation with seed dormancy for NIL*DOG3* and NIL*DOG6* (Fig. 6). However, the response of seed longevity to light was much more pronounced than that of dormancy. Light intensity significantly affected seed longevity for all genotypes tested (Fig. 6B and Supplementary Figure S2).

Our results show that the low maturation temperature significantly increased dormancy in NIL*DOG1* (Fig. 6C), but also in the other genotypes (Supplementary Figure S2). This can be explained by the functional *DOG1* L*er* and Col alleles that are present in these lines, which is supported by the lack of response in the *dog1* mutants (Supplementary Figure S2). Also for temperature we identified a negative correlation between seed dormancy and seed longevity (Fig. 6C, D).

Nitrate dosage, particularly low nitrate (0mM) during silique formation, increased the dormancy levels of NIL*DOG1* and *cyp707a1-1* (Fig. 6E), but not of *cyp707a2-1* (Supplementary Figure S2). Thus, the loss-of-function mutation in *CYP707A2* leads to a defective response to nitrate and therefore no increase in dormancy.

#### The effect of the parental environment on germination in stress conditions

**Fig. 7.**
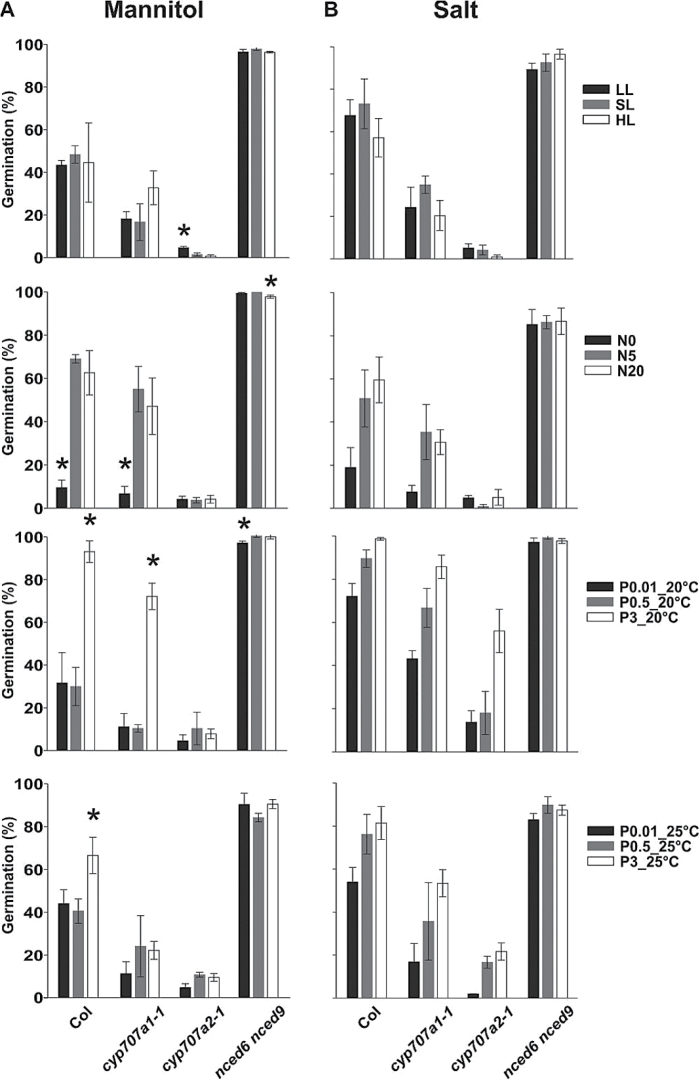
Mannitol (A) and salt (B) stress germination of an ABA biosynthesis double mutant (*nced6 nced9*) and catabolic mutants (*cyp707a1-1* and *cyp707a2-1*) grown under different environments: light intensity [low light (LL), standard light (SL), and high light (HL)], photoperiod [short day (SD), long day (LD) and continuous light (CL)], temperature (15, 20, and 25°C), nitrate concentration (N0, N5, and N20), and phosphate concentration × temperature (P0.01_20°C, P0.5_20°C, P3_20°C, P0.01_25°C, P0.5_25°C, and P3_25°C). Averages of three replicates are presented. Error bars show standard errors. Asterisks indicate significant differences between the treatment and control for each genotype (*P <* 0.05).

In general, high light intensity, continuous light, high temperature (25°C), high nitrate, and high phosphate resulted in higher germination percentage under stress (Supplementary Figure S2). Germination behaviour in mannitol and salt were positively correlated as described above (Fig. 2 and Supplementary Table S2; *P* = 4.05^–96^). The *nced6 nced9* double mutant that produces less ABA in its seeds (Lefebvre *et al.*, 2006) showed the most distinguishable germination phenotype, germinating to approximately 100% in both salt and mannitol, irrespective of the maturation environment (Fig. 7). Meanwhile, *cyp707a2-1* was always sensitive to stress conditions, and to a higher extent than *cyp707a1-1* (Fig. 7).

## Discussion

Knowledge about the effect of the parental environment on seed performance provides more insight into the fundamental principles of how the environment may influence the fitness of a species, as measured by fruit and seed yield, and seed performance. Such knowledge will not only help to predict seed performance but also assist in the improvement of breeding programmes and seed production by indicating the best location, season, and soil type to increase yield and seed quality. By using 12 genotypes and 13 different seed maturation environments, our study provides a very detailed insight into the effect of the environment, genotype, and genotype-by-environment interactions on plant and seed performance.

### Genotype-by-environment interactions

**Fig. 8.**
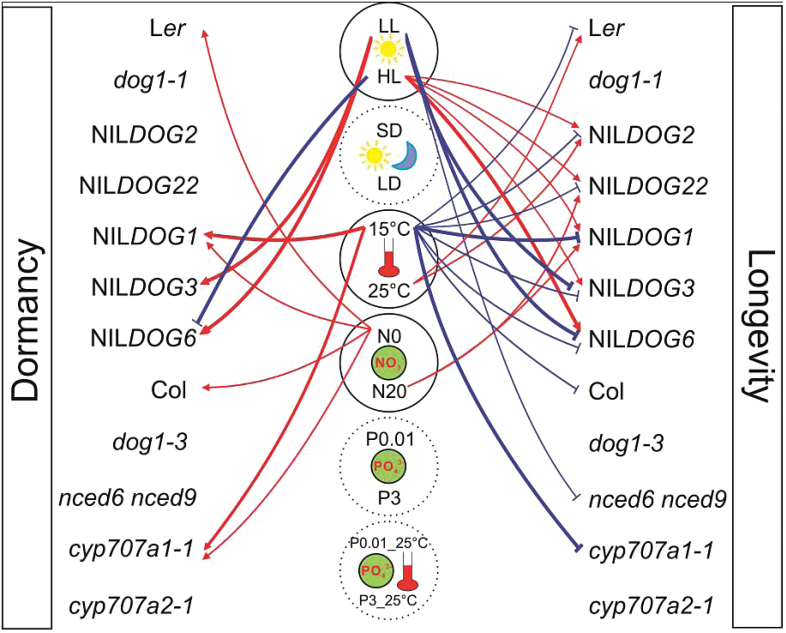
Summarizing model of parental environmental effects on seed dormancy and longevity. Red and blue lines represent the environmental conditions which increase or decrease the trait level, respectively. Bold lines show the negative correlation between dormancy and longevity.

The usefulness of studying different genotypes became apparent from the fact that clear genotype-specific effects were observed. Most obvious is the effect of the genetic background (Table 2, *P <* 1×10^–113^). L*er* and Col genotypes responded with a similar trend to the changes in the environment but Col plants are taller, produce more siliques per plant, and are generally more stress tolerant (higher germination in ABA and higher germination after artificial ageing) (Figs 3 and 4). Furthermore, strong genotype-by-environment interactions were observed (Table 4, Fig. 8). These interactions show that certain genotypes respond differently to specific environmental cues, as discussed below in detail. Similar effects were, to some extent, also shown by Munir *et al.* (2001) who reported a significant genotype-by-maternal photoperiod interaction for Calver-0 and Tacoma-0 recombinant inbred lines in long day and short day conditions. A similar interaction was found in this population for quantitative trait loci (QTL) identified on chromosome 3 which had a significantly stronger effect on fitness in June-dispersed seeds than in November-dispersed seeds (Huang *et al.*, 2010). The role of the maternal photoperiod on genotype-by-environment interactions was also shown for L*er* and Col when combining photoperiod and temperature (Donohue *et al.*, 2007; Donohue, 2009). Contrary to these studies we chose a more comprehensive approach by using a large set of genotypes (12), and measuring five plant and 13 seed performance traits after plant exposure to different light intensities, photoperiods, temperatures, and nitrate and phosphate concentrations.

Photoperiod, phosphate, and phosphate × temperature combinations did not have significant effects on seed dormancy and longevity while light intensity, temperature, and nitrate showed clear genotype-specific responses (Fig. 8). Low light conditions increased dormancy in NIL*DOG3* and NIL*DOG6*, whereas temperature mainly affected NIL*DOG1* and *cyp707a1-1* (Figs 6 and 8). It remains unclear how light intensities can affect NIL*DOG3* and NIL*DOG6* since the underlying genes have not yet been cloned. For *DOG1* it is known that low temperature during seed maturation increases its expression and thereby seed dormancy (Chiang *et al.*, 2011; Kendall *et al.*, 2011; Nakabayashi *et al.*, 2012). This effect of seed maturation temperatures on seed dormancy levels has also recently been reported by Huang *et al.* (2014). Low nitrate conditions also specifically increased seed dormancy in NIL*DOG1* and *cyp707a1-1*, as well as in the background accessions L*er* and Col (Fig. 8). The precise effect of the environment on *DOG1* and *CYP707A1* remains to be investigated. However, both *DOG1* expression and ABA levels in buried seeds increase in winter (Footitt *et al.*, 2011) and *CYP707A1* is probably required for the ABA breakdown. This agrees with the observed higher ABA levels in NIL*DOG1* and *cyp707a1-1* in low nitrate and low temperature conditions (Supplementary Figure S3; also see the ‘Supplementary data’ section for the ABA extraction and detection method).

ABA metabolism and signalling are responsive to many important developmental processes and environmental cues, which makes it a key regulator of growth in changing environments (Nambara and Kuchitsu, 2011). These physiological processes are primarily regulated by ABA maintenance, through fine-tuning of the rates of *de novo* biosynthesis and catabolism (Saito *et al.*, 2004). The ABA biosynthesis-defective *nced6 nced9* double mutant was far less sensitive to changing environments, suggesting a role for *de novo* ABA synthesis during imbibition in these conditions. The expression of the ABA catabolic gene *CYP707A2* was induced dramatically after 6h of imbibition whereas *CYP707A1* did not peak at the early stages of germination. Therefore, *CYP707A2* is probably more effective in the upregulation of ABA degradation during germination (Liu *et al.*, 2009). This hypothesis is supported by the observation that *cyp707a2-1* was always more sensitive to germination under stress (mannitol and salt) than *cyp707a1-1* (Fig. 7 and Supplementary Figure S2).

The responses discussed above are direct effects of the environment on seed development and maturation; however, there might also be indirect responses. The increased length of the reproductive period at low temperature (Fig. 1) can be the cause of the heavier (Fig. 3D) and larger (Fig. 3E) seeds. Seeds are on the plant longer, thus allowing increased nutrient translocation and reserve accumulation. Effects on seed performance in low light intensity, short days, and 15°C seem to be direct since the reproductive period was extended by 10–15 d for all three treatments but seed performance responses were different among the three environments (Fig. 4).

### Negative correlation between seed dormancy and longevity

The negative correlation that we found between seed dormancy and seed longevity is in agreement with the recently reported negative correlation of seed dormancy and seed longevity QTLs in *Arabidopsis* (Nguyen *et al.*, 2012). This surprising finding was in contradiction with all earlier reported work. We hypothesized that this observation was linked to the fact that we used natural variation for our studies, in contrast to the earlier work that was all based on mutant analyses in *Arabidopsis* [i.e. *leafy cotyledon1* (*lec1*), *abscisic acid intensitive3* (*abi3*), *transparent testa* (*tt*), *aberrant testa shape* (*ats*), *delay of germination1* (*dog1*), and the green seed mutant (Ooms *et al.*, 1993; Debeaujon *et al.*, 2000; Clerkx *et al*., 2003, 2004; Bentsink *et al.*, 2006; Sugliani *et al.*, 2009)]. A negative correlation between seed dormancy and seed longevity, confirming our hypothesis, and a role for environmental adaptation has recently been described for *Eruca sativa* (Barazani *et al.*, 2012; Hanin *et al.*, 2013). These authors studied *E. sativa* plants that are distributed in Israel in a narrow geographic area, along different habitats ranging from arid-dry environments to more mesic habitats and found that dormancy increased with increasing aridity and that seed longevity decreased along this gradient. Moreover, De Cauwer *et al.* (2013) have demonstrated variation for dormancy and longevity in natural populations of *Galinsoga parviflore* and *G. quadriradiate*. Also in this weed the less dormant species showed the highest longevity.

A novel aspect of our current work is that we can affect this correlation by changing the seed maturation environment. Increasing light intensity and temperature decreased primary dormancy and increased seed longevity, as measured by artificial ageing (Figs 6 and 8). This may refer to the selective pressure that has shaped natural variation for these traits and thereby their evolutionary path. This is in line with the fact that seed dormancy is an adaptive trait (Bewley, 1997) that displays strong adaptive plasticity to geographic locations and seasonal conditions (Donohue *et al.*, 2005b). Based on our data it is unclear which of the two traits (dormancy or longevity) is under selective pressure since there might be a trade-off between them. Identifying this trade-off mechanism will require more in-depth studies but we speculate that this trade-off represents an adaptive mechanism to maximize fitness under contrasting environments. *Arabidopsis* growing in temperate climates (mild and wet summer conditions) are best synchronized with dormancy cycling mechanisms. These mechanisms involve de- and rehydration cycles allowing, at intervals, activation of repair mechanisms to counteract aging-related damage, such as the repair of damaged DNA, proteins, and mobilization of proteins (Gamboa‐deBuen *et al.*, 2006; Kranner *et al.*, 2010; Rajjou *et al.*, 2012). In warmer and drier climates seeds will have to survive hot and dry conditions. Under these conditions, de- and rehydration cycles are absent and dormancy cycling may be less relevant for fitness. In this case the selective pressure could be on longevity rather than on dormancy. Thus, in high maternal temperature and light, dormancy levels are low because these climatic conditions (seasons) are associated with drought that does not allow germination until water is present. Hence, in this situation germination is controlled by the environment rather than by seed dormancy. The conditions that are encountered by the plant and seeds also depend on whether plants behave like summer or winter annuals, which in addition to germination phenology is determined by flowering time. Analyses of natural populations for which geographical and environmental data is available might provide more insight into the selective pressures that shaped natural variation for these important seed traits (Mendez-Vigo *et al.*, 2011).

### Transfer of knowledge to crops

In the present study we have investigated the effect of single environments and only in one case the effect of a combined environment (phosphate and high temperature) which did not have any significant effect on either plant or seed performance (Table 3), whereas the single environments phosphate and temperature did have significant effects. In the field, environmental conditions are more complex. Plants often have to deal with combinatorial changes. For example, high light intensity is most likely accompanied by high temperature, whereas winter combines cold, short photoperiod, and low radiation. Moreover, our analysis revealed that light intensity may significantly affect plant performance. Doubling the light intensity increased plant height, number of siliques per plant, number of seeds per silique, seed weight, and seed size. Standard laboratory light intensities used in this and other studies (150 μmol m^–2^ s^–1^) are still rather low compared to that of sunlight (in open field, 1500–2000 μmol m^–2^ s^–1^ on sunny days and 200–450 μmol m^–2^ s^–1^ on cloudy days at noon in summer in the Netherlands (Global radiation data in 2012 from De Kring – Bleiswijk, the Netherlands) (Mishra *et al.*, 2012). Our study indicates that *Arabidopsis* benefits substantially from growth at high light intensities. Whether crop plants respond in a similar way remains to be investigated, as well as whether striving for higher sunlight levels has the same advantages. Furthermore, we see that increased photoperiod, especially continuous light, enhances plant performance, which might be detrimental to some crops, such as tomato (Velez-Ramirez *et al.*, 2011). The research performed here in the model plant *Arabidopsis* provides directions for further investigations in crop species.

In general, a combination of low seed dormancy and high longevity is a desirable trait for crop species but also for seed conservation, although a certain level of dormancy may be required to prevent pre-harvest sprouting. Our study implies that manipulation of the growth conditions may be used to culture seeds with the required seed performance. Alternatively, monitoring the growth conditions during seed maturation may help to predict performance of the mature seed.

In conclusion, our comparative analyses confirm and extend the notion that variable environmental conditions during seed development may result in variable plant and seed performance. Of all five parental environments analysed, temperature changes during seed maturation played a dominant role in both plant and seed performance, whereas light signalling (light intensity and photoperiod) had more impact on plant traits. Nitrate and phosphate displayed relatively mild effects on plant and seed performance. The observation that the different genotypes responded differentially to the environmental conditions indicates that different genetic and molecular pathways are involved in these responses.

## Supplementary material

Supplementary data can be found at *JXB* online, and includes the ABA extraction and detection method as well as the following:

Supplementary Table S1. Element concentrations in the standard nutrient solution

Supplementary Table S2. Trait by trait correlation/significance of plant and seed performance.

Supplementary Figure S1. Seed dormancy and longevity levels of plants grown in control conditions (i.e. standard light, long days, 20°C, 5mM nitrate and 0.5mM phosphate).

Supplementary Figure S2. Plant and seed performances of each genotype in five environments (light intensity, photoperiod, temperature, nitrate, and phosphate).

Supplementary Figure S3. ABA levels in freshly harvested seeds of L*er*, NIL*DOG1*, Col, and *cyp707a1-1* matured in low temperature and low nitrate compared with the control conditions.

Supplementary Data
