# Supplementary material for: Interaction between parental environment and genotype affects plant and seed performance in Arabidopsis
Source: J Exp Bot. 2014 Sep 18;65(22):6603–15. doi: 10.1093/jxb/eru378 (PMC4246189; doi:10.1093/jxb/eru378)
Supplement: Supplementary Data [file supp_65_22_6603__index.html]

Interaction between parental environment and genotype affects plant and seed performance in Arabidopsis — Interaction between parental environment and genotype affects plant and seed performance in Arabidopsis — Supplementary Data 

# Interaction between parental environment and genotype affects plant and seed performance in *Arabidopsis*

## Supplementary Data

Data files

**Files in this Data Supplement:**

- Supplementary Data - Supplementary Data
