# Supplementary material for: Interaction between parental environment and genotype affects plant and seed performance in Arabidopsis
Source: J Exp Bot. 2014 Sep 18;65(22):6603–15. doi: 10.1093/jxb/eru378 (PMC4246189; doi:10.1093/jxb/eru378)
Supplement: Supplementary Data [file supp_eru378_jexbot131375_file001.pdf]

Supplementary Data:

Journal of Experimental Botany

Title:Interaction between parental environment and genotype affects plant and seed performance in Arabidopsis

Authors: Hanzi He[1]*, Deborah de Souza Vidigal[1]*, L. Basten Snoek[3], Sabine Schnabel[4,5], Harm Nijveen[6], Henk Hilhorst[1], Leónie Bentsink[1,2]

Table S1. Element concentrations in the standard nutrient solution

	Macro-element (mM)								Micro-element (μM)					
element	N	K	Ca	Na	Mg	Cl	S	P	Fe	Mn	Zn	B	Cu	Mo
concentration	5	2.9	2	0.4	0.5	0.4	1.1	0.5	3.5	2.6	3.0	20	6.6	0.2

The pH of the solution was 7.0

Table S2. Trait by trait correlation/significance of plant and seed performance. The linear model was used to calculate all pairwise correlations between plant and seed performance traits. Pearson correlation coefficient r values are presented in the lower diagonal, corresponding to red/blue rectangles in Fig. 2. In the yellow/grey colored area, rectangles represent –log (*P*-values) respective to the Pearson correlation coefficients are presented in the upper diagonal, corresponding to yellow/grey rectangles in Figure 2. DSDS50 (days of seed dry storage until 50% of germination) represents dormancy levels. Longevity is measured by artificial aging (40°C, 85% relative humidity). Gmax is the final germination percentage at the end of the germination assay. t50 is the rate of germination. NA: not available.

4.01	17.25	4.36	26.51	5.23	0.25	8.66	0.70	1.96	4.67	2.21	21.51	2.62	3.79	0.44	NA	1.72	-	Phytate content
0.67	2.67	1.21	13.73	2.44	1.77	12.24	1.01	15.40	0.52	23.53	3.57	7.67	4.21	13.07	NA	-	-0.11	Phosphate content
0.49	1.19	3.13	0.98	1.29	0.88	0.54	0.13	0.25	2.61	0.03	0.02	1.54	0.76	0.33	-	NA	NA	Nitrate content
20.07	31.63	3.32	11.68	8.10	3.53	13.65	2.94	2.04	0.10	1.79	9.32	25.86	3.93	-	0.06	0.35	-0.04	Gmax ABA
5.68	5.99	1.75	1.90	3.01	47.24	23.67	30.54	56.32	11.13	24.39	92.63	95.39	-	0.11	0.11	0.19	0.18	Gmax salt
5.90	0.61	1.03	0.20	1.86	36.58	0.30	23.09	29.64	5.14	6.56	56.80	-	0.55	0.30	0.18	0.27	0.15	Gmax mannitol
4.01	1.53	11.36	6.33	4.94	26.10	5.19	54.22	29.82	19.13	5.73	-	0.43	0.54	0.18	0.00	-0.17	0.44	Gmax 30°C
42.03	0.53	2.48	7.39	13.83	21.95	59.20	15.92	126.39	35.70	-	0.14	0.15	0.29	0.07	0.01	0.46	-0.13	t50 10°C
4.52	0.09	9.84	0.12	2.27	1.47	33.87	93.93	24.77	-	0.35	0.26	0.13	0.19	-0.01	-0.25	-0.05	0.20	Gmax 10°C
21.95	0.48	2.51	1.11	7.78	53.36	32.91	28.26	-	0.29	0.61	0.32	0.32	0.43	0.07	0.05	0.38	-0.12	t50 22°C
0.58	1.28	2.50	0.03	0.15	6.15	14.78	-	0.31	0.54	0.23	0.42	0.28	0.32	0.09	0.03	0.08	-0.06	Gmax 22°C
30.17	6.31	0.86	27.42	15.33	3.31	-	0.22	0.34	0.34	0.44	0.13	0.02	0.29	0.22	-0.09	0.34	-0.28	Longevity
0.31	0.54	1.05	0.06	0.86	-	-0.10	-0.14	-0.42	0.06	-0.27	-0.30	-0.35	-0.40	-0.10	-0.13	-0.11	0.03	DSDS50
58.38	18.05	0.32	52.41	-	-0.04	0.23	-0.01	0.16	0.08	0.22	-0.12	-0.07	-0.09	0.16	-0.16	-0.14	-0.22	Seed weight
19.33	25.92	1.29	-	0.41	0.00	0.31	0.00	0.05	-0.01	0.16	-0.14	0.01	-0.07	0.20	-0.14	0.36	-0.49	Seed size
3.80	27.12	-	0.06	0.02	0.05	0.05	0.09	-0.09	0.20	-0.09	0.22	0.05	-0.07	0.11	0.28	0.13	0.27	Seeds per silique
133.42	-	0.35	0.31	0.26	0.03	0.16	-0.06	-0.03	0.01	0.03	-0.07	-0.04	-0.15	0.36	0.15	-0.15	0.40	Silique per plant
-	0.65	0.13	0.27	0.46	0.02	0.35	-0.04	0.30	0.13	0.41	-0.12	-0.15	-0.15	0.29	-0.08	-0.06	0.19	Plant height
Plant height	Silique per plant	Seeds per silique	Seed size	Seed weight	DSDS50	Longevity	Gmax 22°C	t50 22°C	Gmax 10°C	t50 10°C	Gmax 30°C	Gmax mannitol	Gmax salt	Gmax ABA	Nitrate content	Phosphate content	Phytate content	

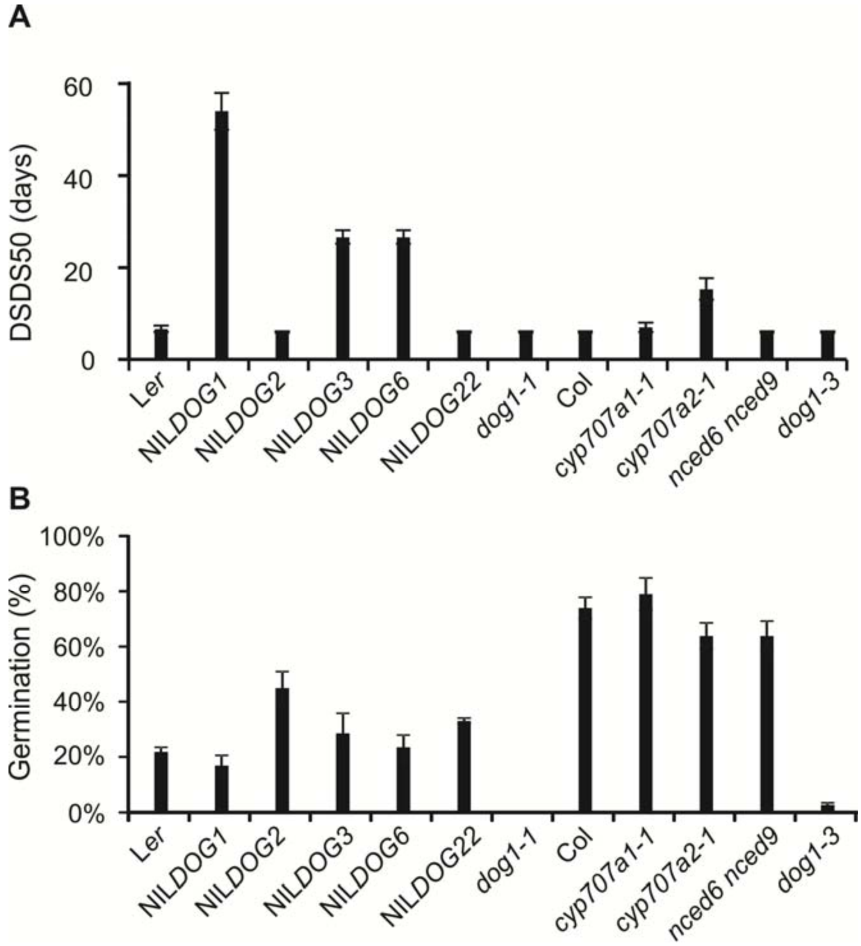

Figure S1. Seed dormancy levels (A) and seed longevity levels (B) of plants grown in control condition (i.e. standard light (SL), long day (LD), 20°C, nitrate 5 mM (N5), phosphate 0.5 mM (P0.5)).

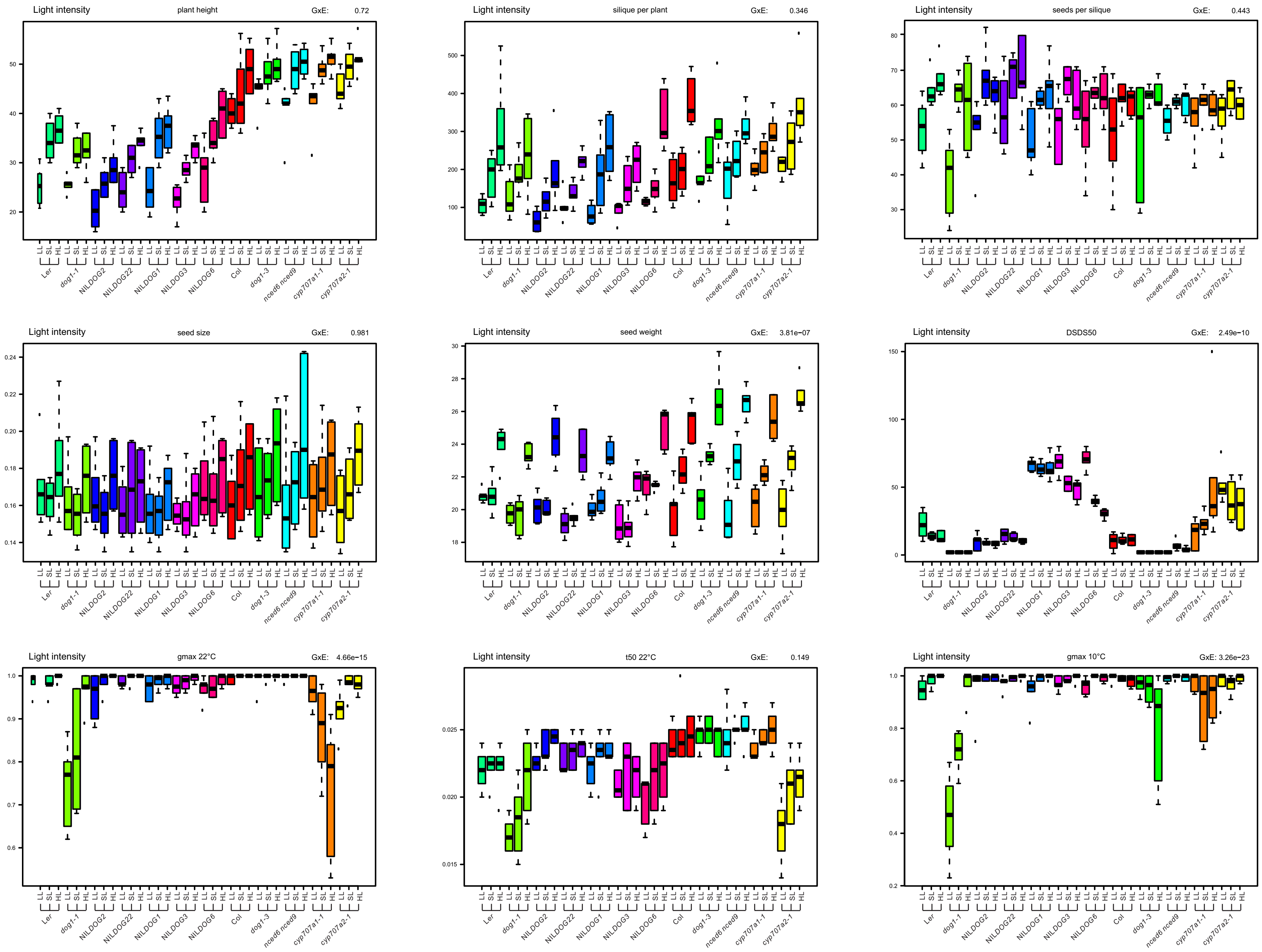
Light intensity
plant height
GxE: 0.72
silique per plant
GxE: 0.346
seeds per silique
GxE: 0.443
seed size
GxE: 0.981
seed weight
GxE: 3.81e−07
DSDS50
GxE: 2.49e−10
gmax 22°C
GxE: 4.66e−15
t50 22°C
GxE: 0.149
gmax 10°C
GxE: 3.26e−23
LL SL HL
Ler
dog1-1
NILDOG2
NILDOG22
NILDOG1
NILDOG3
NILDOG6
Col
dog1-3
nced6 nced9
cyp707a1-1
cyp707a2-1

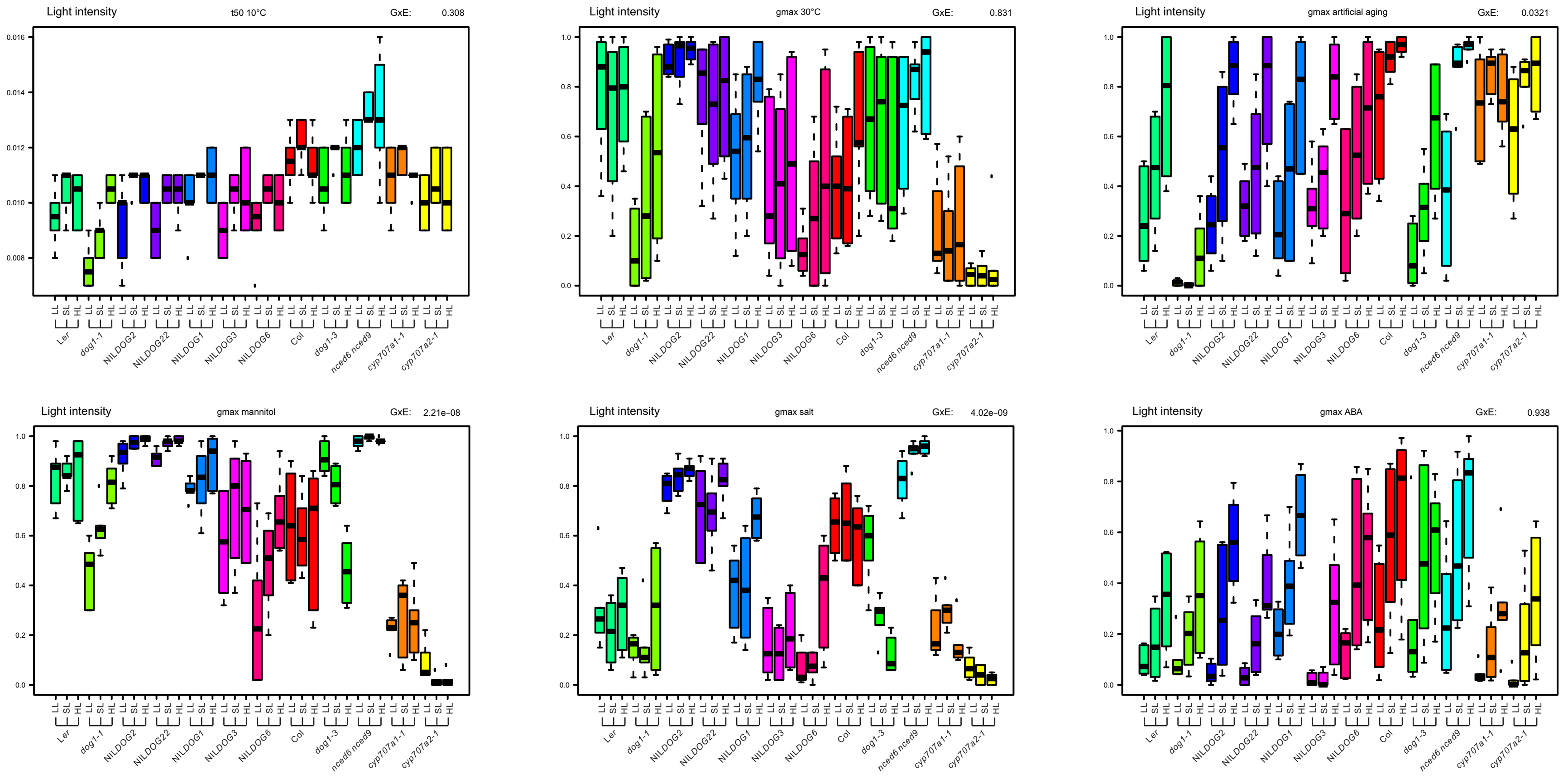
Light intensity
t50 10°C
GxE: 0.308
Light intensity
gmax 30°C
GxE: 0.831
Light intensity
gmax artificial aging
GxE: 0.0321
Light intensity
gmax mannitol
GxE: 2.21e−08
Light intensity
gmax salt
GxE: 4.02e−09
Light intensity
gmax ABA
GxE: 0.938
LL SL HL
Ler
dog1-1
NILDOG2
NILDOG22
NILDOG1
NILDOG3
NILDOG6
Col
dog1-3
nced6 nced9
cyp707a1-1
cyp707a2-1

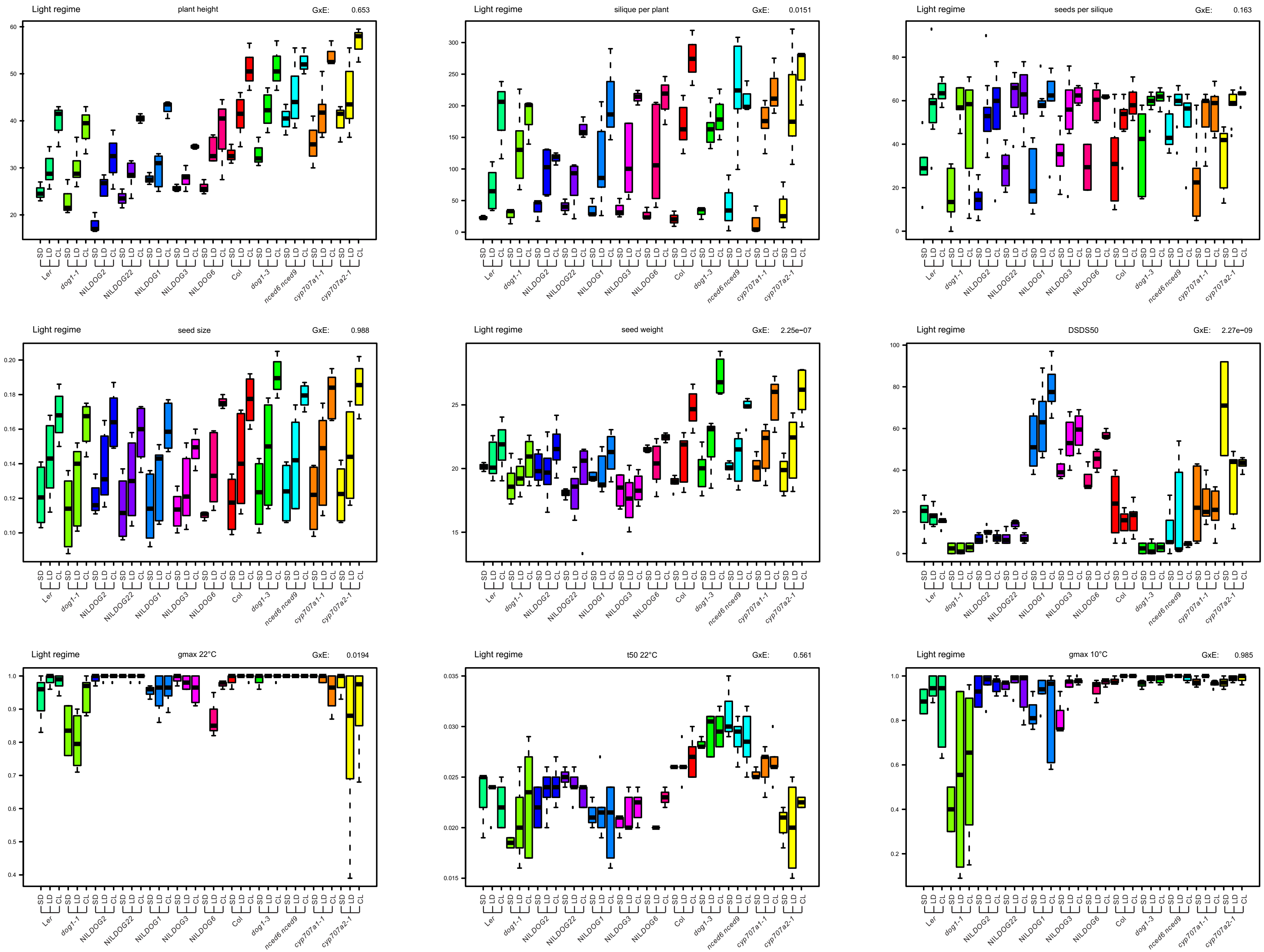
Light regime
plant height
GxE: 0.653
silique per plant
GxE: 0.0151
seeds per silique
GxE: 0.163
seed size
GxE: 0.988
seed weight
GxE: 2.25e−07
DSDS50
GxE: 2.27e−09
gmax 22°C
GxE: 0.0194
t50 22°C
GxE: 0.561
gmax 10°C
GxE: 0.985
SD LD CL
Ler
dog1-1
NILDOG2
NILDOG22
NILDOG1
NILDOG3
NILDOG6
Col
dog1-3
nced6 nced9
cyp707a1-1
cyp707a2-1

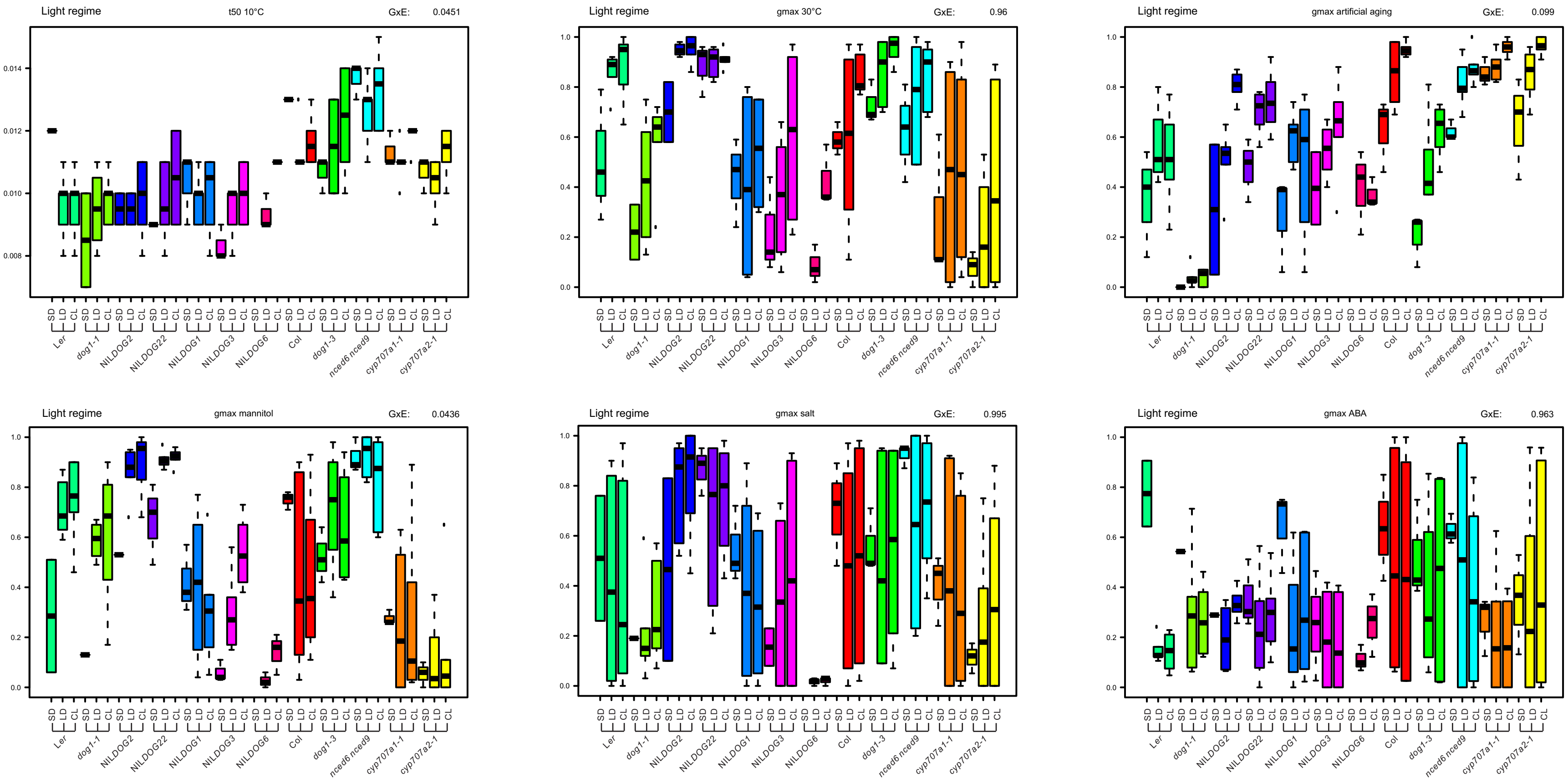
Light regime
t50 10°C
GxE: 0.0451
Light regime
gmax 30°C
GxE: 0.96
Light regime
gmax artificial aging
GxE: 0.099
Light regime
gmax mannitol
GxE: 0.0436
Light regime
gmax salt
GxE: 0.995
Light regime
gmax ABA
GxE: 0.963
SD LD CL
Ler
dog1-1
NILDOG2
NILDOG22
NILDOG1
NILDOG3
NILDOG6
Col
dog1-3
nced6 nced9
cyp707a1-1
cyp707a2-1

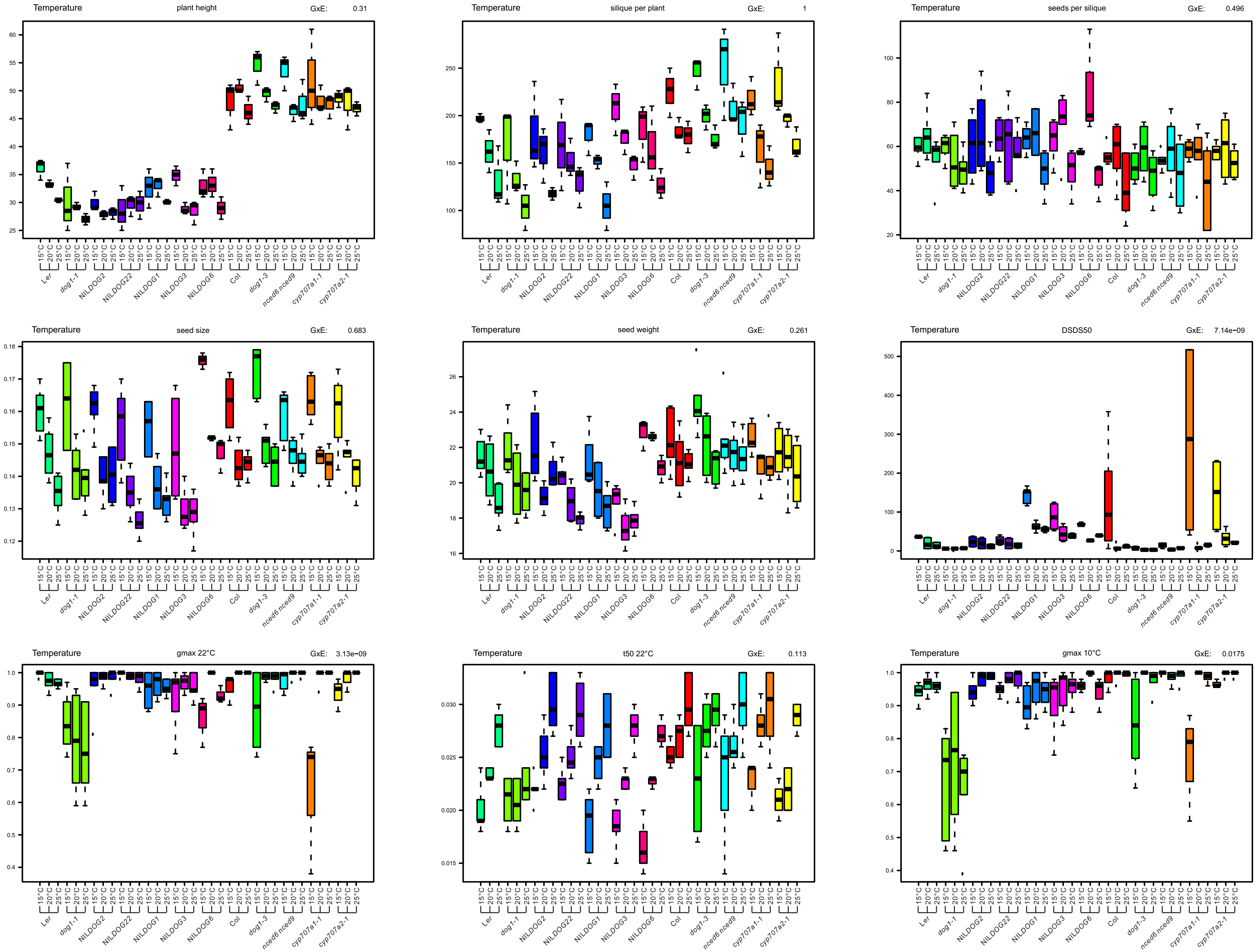

Temperature
plant height
GxE: 0.31
Temperature
silique per plant
GxE: 1
Temperature
seeds per silique
GxE: 0.496
Temperature
seed size
GxE: 0.683
Temperature
seed weight
GxE: 0.261
Temperature
DSDS50
GxE: 7.14e−09
Temperature
gmax 22°C
GxE: 3.13e−09
Temperature
t50 22°C
GxE: 0.113
Temperature
gmax 10°C
GxE: 0.0175
15°C
20°C
25°C
Ler
dog1-1
NILDOG2
NILDOG22
NILDOG1
NILDOG3
NILDOG6
Col
dog1-3
nced6 nced9
cyp707a1-1
cyp707a2-1

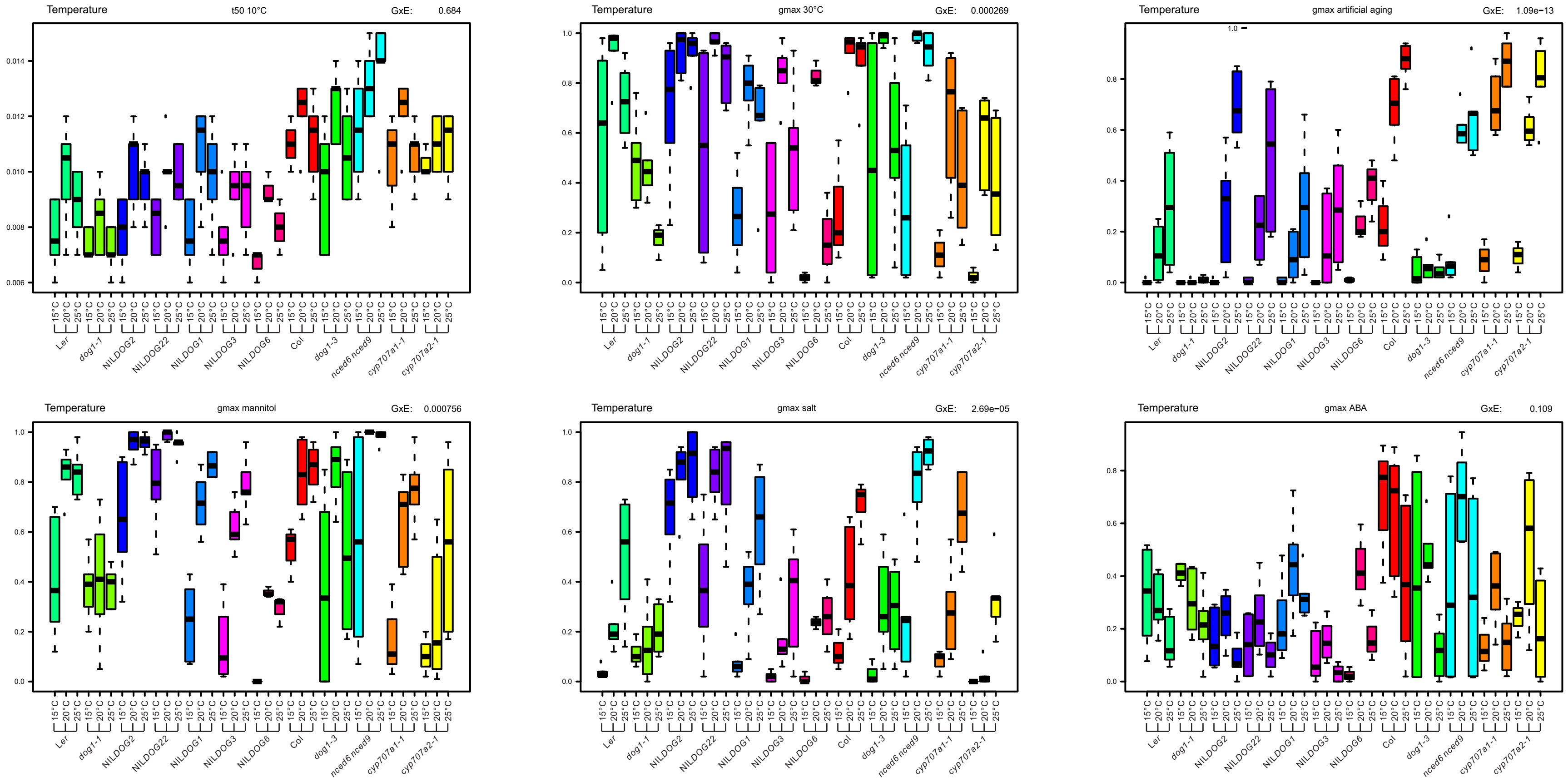
Temperature
t50 10°C
GxE: 0.684
Temperature
gmax 30°C
GxE: 0.000269
Temperature
gmax artificial aging
GxE: 1.09e−13
Temperature
gmax mannitol
GxE: 0.000756
Temperature
gmax salt
GxE: 2.69e−05
Temperature
gmax ABA
GxE: 0.109
15°C 20°C 25°C
Ler
dog1-1
NILDOG2
NILDOG22
NILDOG1
NILDOG3
NILDOG6
Col
dog1-3
nced6 nced9
cyp707a1-1
cyp707a2-1

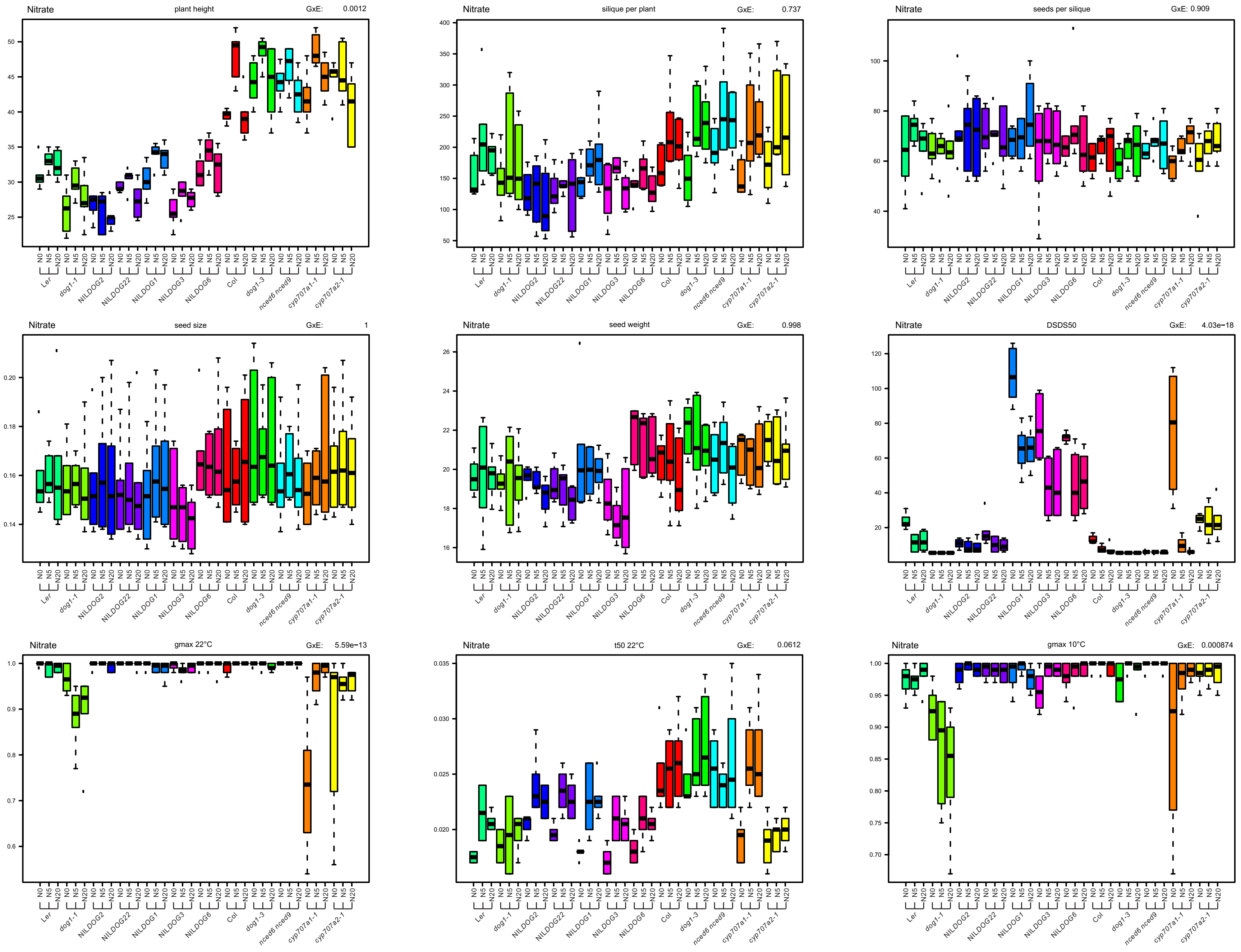
Nitrate
plant height
GxE: 0.0012
silique per plant
GxE: 0.737
seeds per silique
GxE: 0.909
seed size
GxE: 1
seed weight
GxE: 0.998
DSDS50
GxE: 4.03e−18
gmax 22°C
GxE: 5.59e−13
t50 22°C
GxE: 0.0612
gmax 10°C
GxE: 0.000874
N0 N5 N20
Ler
dog1-1
NILDOG2
NILDOG22
NILDOG1
NILDOG3
NILDOG6
Col
dog1-3
nced6 nced9
cyp707a1-1
cyp707a2-1

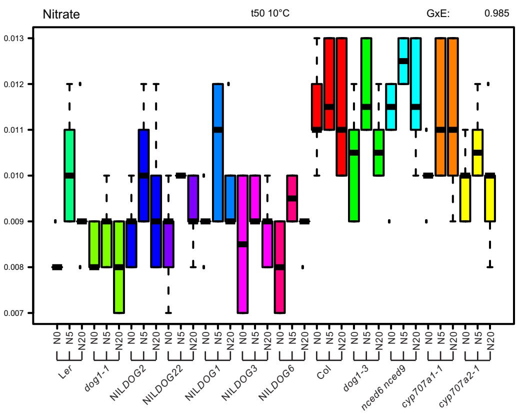
Nitrate
t50 10°C
GxE:
0.985
N0 N5 N20
Ler dog1-1 NILDOG2 NILDOG22 NILDOG1 NILDOG3 NILDOG6 Col dog1-3 nced6 nced9 cyp707a1-1 cyp707a2-1

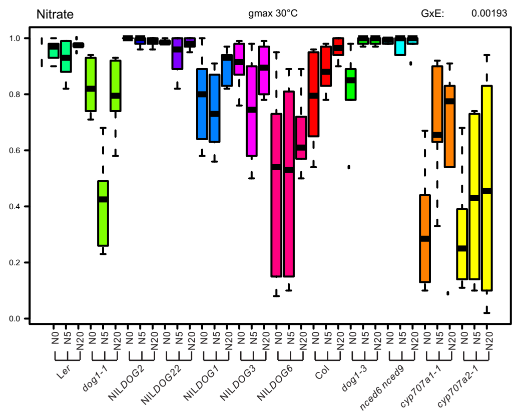
Nitrate
gmax 30°C
GxE:
0.00193
N0 N5 N20
Ler dog1-1 NILDOG2 NILDOG22 NILDOG1 NILDOG3 NILDOG6 Col dog1-3 nced6 nced9 cyp707a1-1 cyp707a2-1

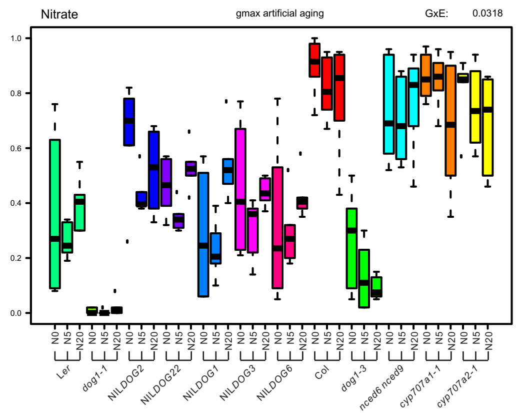
Nitrate
gmax artificial aging
GxE:
0.0318
N0 N5 N20
Ler dog1-1 NILDOG2 NILDOG22 NILDOG1 NILDOG3 NILDOG6 Col dog1-3 nced6 nced9 cyp707a1-1 cyp707a2-1

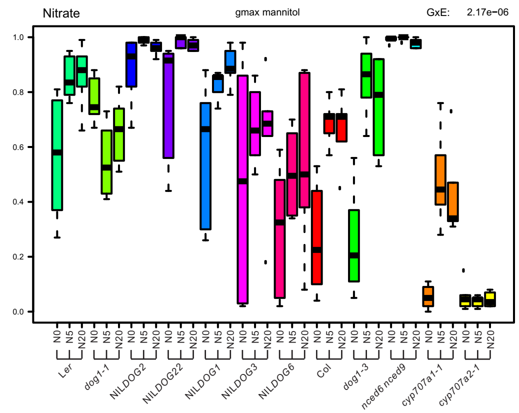
Nitrate
gmax mannitol
GxE:
2.17e−06
N0 N5 N20
Ler dog1-1 NILDOG2 NILDOG22 NILDOG1 NILDOG3 NILDOG6 Col dog1-3 nced6 nced9 cyp707a1-1 cyp707a2-1

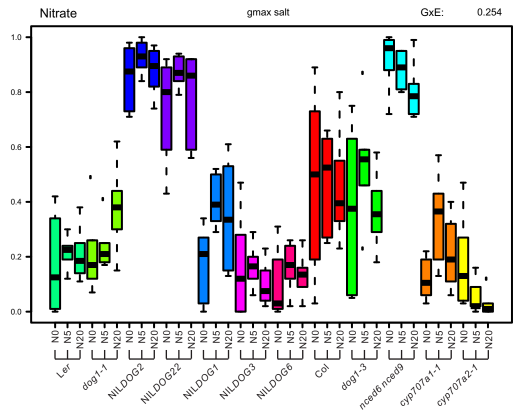
Nitrate
gmax salt
GxE:
0.254
N0 N5 N20
Ler dog1-1 NILDOG2 NILDOG22 NILDOG1 NILDOG3 NILDOG6 Col dog1-3 nced6 nced9 cyp707a1-1 cyp707a2-1

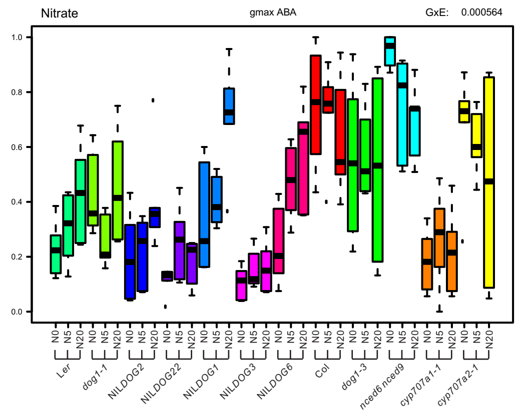
Nitrate
gmax ABA
GxE:
0.000564
N0 N5 N20
Ler dog1-1 NILDOG2 NILDOG22 NILDOG1 NILDOG3 NILDOG6 Col dog1-3 nced6 nced9 cyp707a1-1 cyp707a2-1

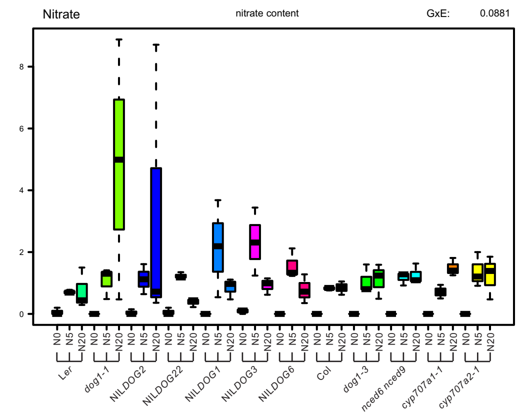
Nitrate
nitrate content
GxE:
0.0881
N0 N5 N20
Ler dog1-1 NILDOG2 NILDOG22 NILDOG1 NILDOG3 NILDOG6 Col dog1-3 nced6 nced9 cyp707a1-1 cyp707a2-1

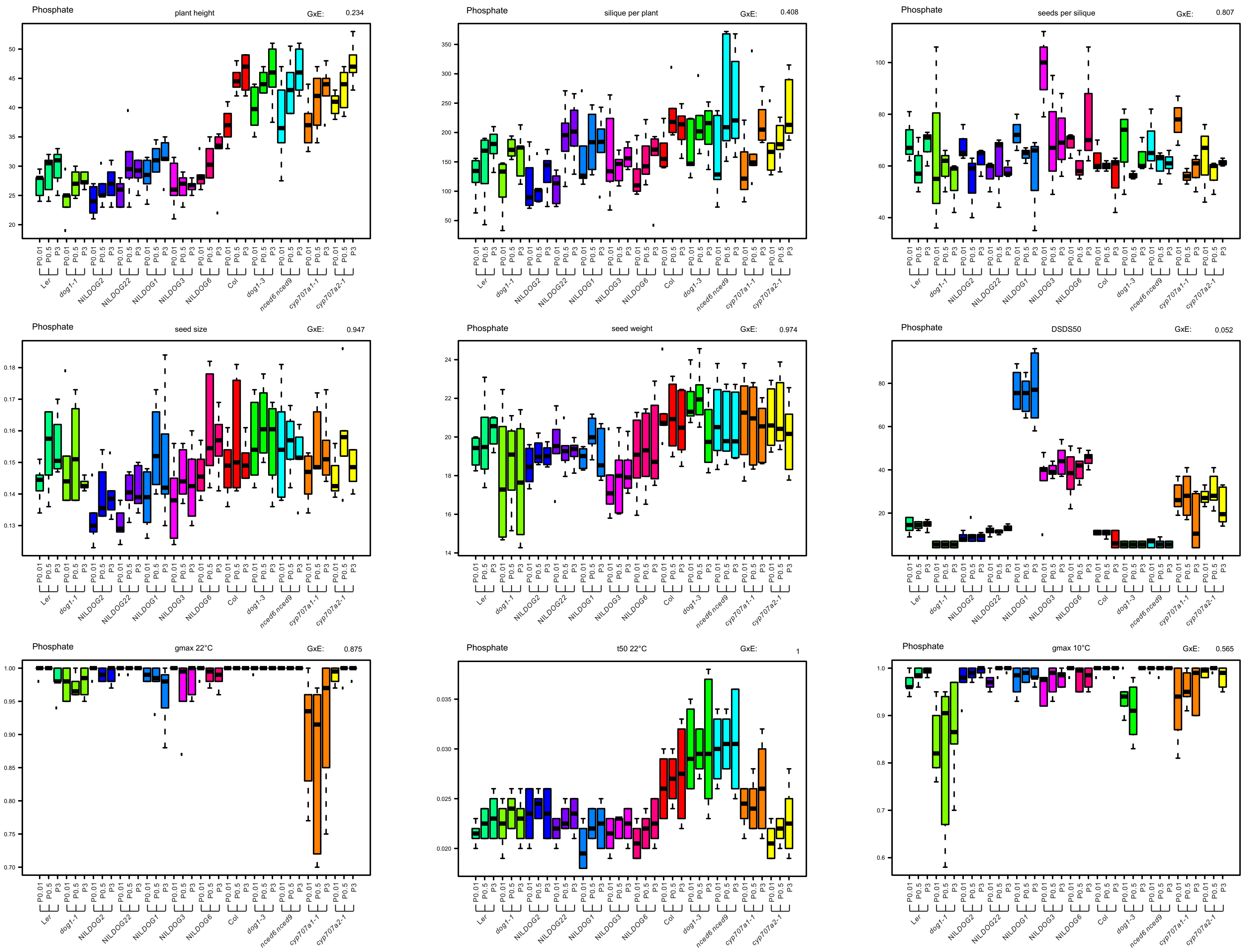
Phosphate
plant height
GxE: 0.234
Phosphate
silique per plant
GxE: 0.408
Phosphate
seeds per silique
GxE: 0.807
Phosphate
seed size
GxE: 0.947
Phosphate
seed weight
GxE: 0.974
Phosphate
DSDS50
GxE: 0.052
Phosphate
gmax 22°C
GxE: 0.875
Phosphate
t50 22°C
GxE: 1
Phosphate
gmax 10°C
GxE: 0.565
P0.01
P0.5
P3
Ler
dog1-1
NILDOG2
NILDOG22
NILDOG1
NILDOG3
NILDOG6
Col
dog1-3
nced6 nced9
cyp707a1-1
cyp707a2-1

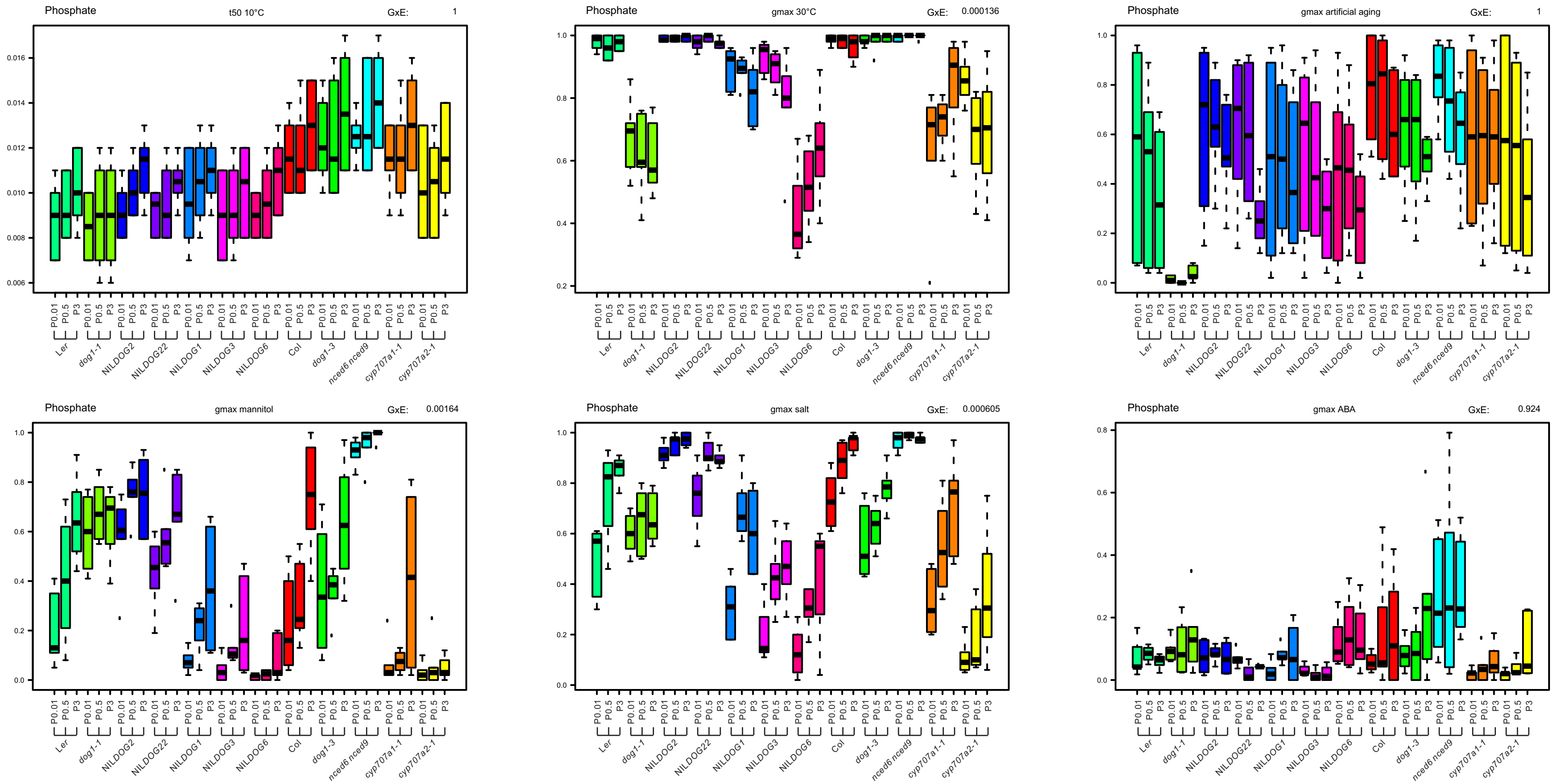

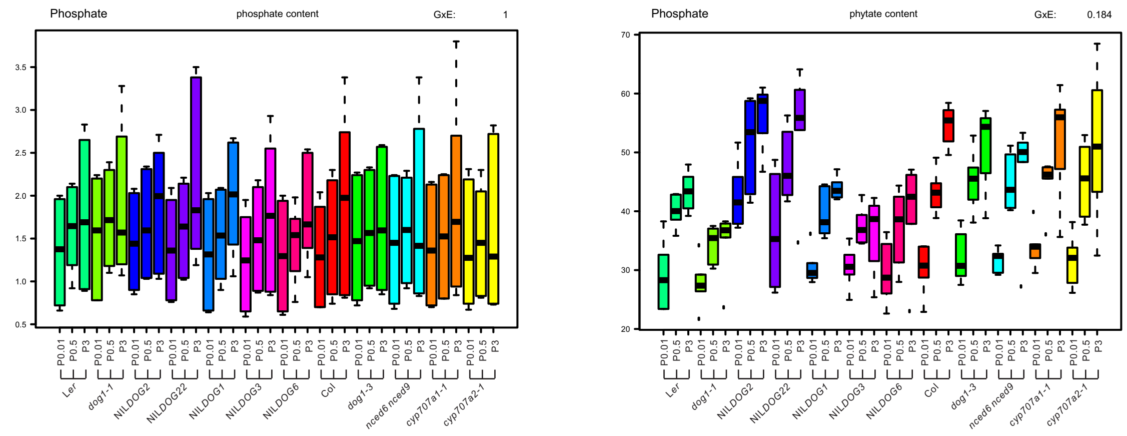

Figure S2: Plant and seed performances of each genotype in five environments (light intensity, light regime, temperature, nitrate and phosphate). Genotype by environment (GxE) interactions are shown in the right corner of each figure.

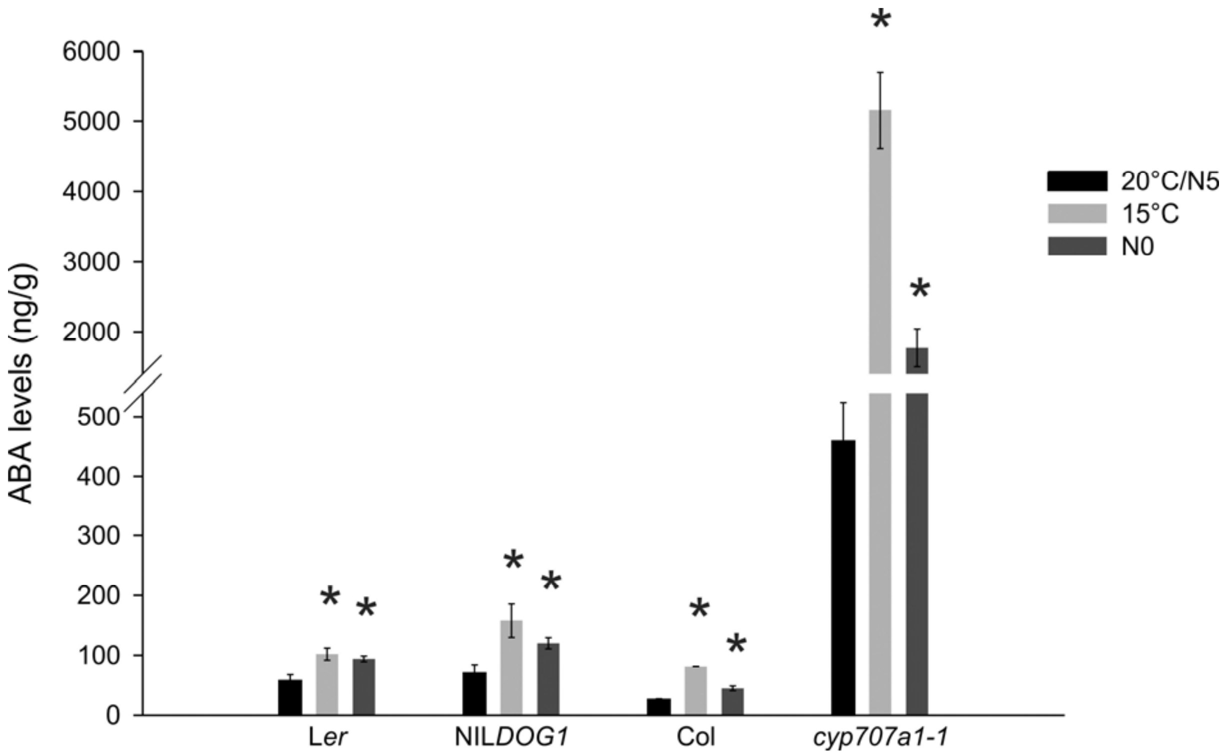

Figure S3. ABA levels in freshly harvested seeds of L*er*, NIL*DOG1*, Col and *cyp707a1-1* matured in low temperature (15°C) and low nitrate (N0) compared with the control condition (20°C/N5). Asterisks represent 0.05 significance between treatment (15°C or N0) and control (20°C/N5)

ABA extraction and detection method

ABA purification was performed by adapting the protocol described by Zhou et al. (2003). 10 mg of dry seeds were frozen in liquid nitrogen and ground with a tissue lyser (Mo Bio Laboratory) at 25 Hz for 1.5 min with help of stainless steel beads. ABA was extracted with 1.5 ml of methanol/water/acetic acid (80:19:1) and 25 pmol ml^{-1} of [2H6]-ABA as internal standard in a 2 ml centrifuge tube. The tubes were vortexed and sonicated for 10 min in a Branson 3510 ultrasonic bath (Branson Ultrasonics, Danbury, CT, US). Samples were centrifuged for 10 min at 2500 g and the liquid phase was carefully transferred to a 4 ml glass vial. The samples were re-extracted with 1.5 ml of methanol/water/acetic acid (80:19:1). Both fractions were combined in a 4 ml glass vial and dried in a speedvac concentrator coupled to a refrigerated vapour trap (Thermo Fisher Scientific). The residue was dissolved in 100 µl methanol/acetic acid 99:1 (v/v) and 900 µl 1% acetic acid in UPLC grade water. Undissolved particles were pelleted before loading the samples on HLB columns (Oasis®, Waters, 30 mg 1 cc) which were previously equilibrated with 1 ml 100% methanol (HPLC supra gradient) followed by 1 ml of methanol/water/acetic acid (10:89:1). The columns/samples were washed with 1 ml methanolwateracetic acid (10:89:1). After washing, 1mL methanol/water/acetic acid (80:19:1) was added to the columns and the flow through was collected. The samples were dried in a speedvac and re-suspended in 100 µl UPLC grade water/acetonitrile/formic acid 94.9:5:0.1. The samples were stored at -20°C until measurement.

ABA analysis was performed with a Waters Xevo tandem quadrupole mass spectrometer equipped with an electrospray ionization source and coupled to an Acquity UPLC system (Waters, USA). Chromatographic separation was achieved using an Acquity UPLC BEH C18 column (100 x 2.1 mm, 1.7 µm) (Waters, USA), applying a water/acetonitrile gradient, starting at 5% acetonitrile for 1.0 min, raised to 98% (v/v) acetonitrile in 5 min which was then maintained for 4 min before returning to 5% acetonitrile in water using a 0.13 min gradient. The column was equilibrated at this solvent composition for 1.87 min prior to the next injection. Total run time was 11 min. The column was operated at 50°C with a flow-rate of 0.2 ml min^{-1} and sample injection volume was 10 µl. The mass spectrometer was operated in positive electrospray ionization (ESI) mode. The cone and desolvation gas flows were 50 and 1000 l h^{-1}, respectively. Argon was used for fragmentation by collision-induced dissociation in the ScanWave collision cell. The capillary voltage was set at 3 kV, the

source temperature at 150°C and the desolvation gas temperature at 550°C. The cone voltage (CV), collision energy (CE) and parent daughter transitions were optimized by injecting pure ABA and [2H6]-ABA in the Waters IntelliStart MS console. Transitions were selected based on the most abundant and specific fragment ions. For ABA, the Multiple reaction monitoring (MRM) transitions m/z 265.16>173.07 at a CE of 18 eV, 265.16>187.05 at 18 eV, 265.16>229.15 at 10 eV and 265.16>247.17 at 6 eV; and for [2H6]-ABA, the transitions m/z 271.22>234.49 at CE of 10 eV, 271.22>253.24 at CE of 6 eV. The cone voltage for all transitions was 10V. ABA was quantified using a calibration curve with known amount of ABA based on the ratio of the summed area of the MRM transitions for ABA to those for [2H6]-ABA. Data acquisition was performed using MassLynx 4.1 software (Waters, USA).

References

Zhou R, Squires TM, Ambrose SJ, Abrams SR, Ross AR, Cutler AJ (2003) Rapid extraction of abscisic acid and its metabolites for liquid chromatography-tandem mass spectrometry. J Chromatogr A 1010: 75-85
